# Endothelial Protein kinase D1 is a major regulator of post-traumatic hyperinflammation

**DOI:** 10.3389/fimmu.2023.1093022

**Published:** 2023-03-02

**Authors:** Jonathan Schönfelder, Tanja Seibold, Mareen Morawe, Robert Sroka, Nora Schneider, Jierui Cai, Josip Golomejic, Lena Schütte, Milena Armacki, Markus Huber-Lang, Miriam Kalbitz, Thomas Seufferlein, Tim Eiseler

**Affiliations:** ^1^ Department of Internal Medicine I, University Hospital Ulm, Ulm, Germany; ^2^ Institute of Clinical and Experimental Trauma-Immunology, University Hospital Ulm, Ulm, Germany; ^3^ Department of Traumatology, Hand-, Plastic, and Reconstructive Surgery, University Hospital Ulm, Ulm, Germany

**Keywords:** trauma, hyperinflammation, sEVs, protein kinase D, leukotriene B4

## Abstract

Trauma is a major cause of death worldwide. The post-traumatic immune response culminates in the release of pro-inflammatory mediators, translating in the infiltration of neutrophils (PMNs) at injury sites. The extent of this inflammation is determined by multiple factors, such as PMN adhesion to the endothelium, transendothelial migration, endothelial barrier integrity as well as PMN swarming, mass infiltration and activation. This process is initiated by secondary lipid mediators, such as leukotriene B_4_ (LTB_4_). We here provide evidence that Protein kinase D1 (PRKD1) in endothelial cells is implicated in all these processes. Endothelial PRKD1 is activated by pro-inflammatory stimuli and amplifies PMN-mediated inflammation by upregulation of cytokine and chemokines as well as adhesion molecules, such as ICAM-1, VCAM-1 and E-selectin. This induces enhanced PMN adhesion and trans-migration. PRKD1 activation also destabilizes endothelial VE-cadherin adhesion complexes and thus the endothelial barrier, fostering PMN infiltration. We even describe a yet unrecognized PRKD1-dependant mechanism to induce biosynthesis of the PMN-swarming mediator LTB_4_ directed *via* intercellular communication through small extracellular vesicles (sEVs) and enhanced CXCL8 secretion from activated endothelial cells. These endothelial sEVs transfer the LTB_4_ biosynthesis enzyme LTA_4_ hydrolase (LTA_4_H) to prime PMNs, while initiating biosynthesis also requires additional signals, like CXCL8. We further demonstrate the respective LTA_4_H-positive sEVs in the serum of polytrauma patients, peaking 12 h post injury. Therefore, PRKD1 is a key regulator in the coordinated communication of the endothelium with PMNs and a vital signaling node during post-traumatic inflammation.

## 1 Introduction

Trauma is the leading cause of death in individuals under 44 years of age (1, 2). One of the most common critical injuries is the isolated chest trauma with a 10–20% mortality rate (3). In patients with multiple injuries a chest trauma increases lethality from 4 to 25%. Immediate and early fatalities after severe trauma are mostly caused by blood loss, brain injuries and critical injuries to vital organs. However, late mortality is also a problem which accounts for 10-30% of trauma-related deaths. It is caused by an imbalanced local and systemic immune reaction (Systemic Inflammatory Response Syndrome, SIRS) (4) and can be accompanied by severe side effects, such as multi organ dysfunction syndrome (MODS), acute respiratory distress syndrome (ARDS) as well as sepsis (5, 6). A systemic immune response after trauma usually starts within 30 min after a major injury and is triggered by the release of danger-associated molecular patterns (DAMPs), originating from damaged or necrotic tissue. DAMPs subsequently induce the rapid recruitment of distinct immune cells, including neutrophil granulocytes and monocytes, accompanied by the activation of complement cascades and the release of inflammatory mediators, like cytokines and chemokines (7). These mediators activate innate immune cells, such as neutrophil (polymorphonuclear leukocytes, PMNs), but also endothelial cells, which in turn produce more inflammatory agents to further attract even more neutrophils to the site of injury (8). The host’s inflammatory response therefore critically relies on the proper interaction between the endothelium and infiltrating PMNs (9) that is controlled at multiple levels. One of them is adhesion to the endothelial surface *via* endothelial cell adhesion molecules, such as E-selectin, Intercellular Adhesion Molecule-1 (ICAM-1) and Vascular cell adhesion protein-1 (VCAM-1), which bind to their corresponding receptors on PMNs (10). Upon adhesion, PMNs actively breach the endothelial barrier. The integrity of the endothelial barrier is a major factor determining the host’s post-traumatic immune response. PMNs transmigrate through the barrier by following chemotactic gradients, like N-formylated tripeptides (Formyl-Methionyl-Leucyl-Phenylalanin, fMLP) (9). In addition, at the site of injury, PMNs further establish secondary gradients, e.g. by Leukotriene B_4_ (LTB_4_) that lure and activate more neutrophils by switching to swarming behavior (11). Swarming is self-organized and the decision to release chemoattractant is critical for the ultimate extent of the post-traumatic immune response (6). However, the exact molecular mechanisms that regulate the complex intercellular exchange between the endothelium and PMNs by direct cell-cell contacts, secretory crosstalk, or extracellular vesicles during infiltration of injuries are still incompletely understood. We have recently shown that the activity of the serine/threonine kinase Protein kinase D1 (PRKD1) controls neutrophil deformability and trans-endothelial migration by modulating actin polymerization and turnover (9). It belongs to a family of three protein kinases with biological functions implicated in cell motility, cell-cell adhesion, transcription, vesicle transport and secretion of different cargos, including small extracellular vesicles (sEVs, exosomes), which are vital messengers of intercellular communication (12, 13). We here provide molecular and functional evidence that PRKD1 is activated in endothelial cells by post-traumatic cytokines, destabilizes the endothelial barrier and induces expression and secretion of neutrophil-relevant pro-inflammatory cytokines, i.e., interleukin-6 (IL6) and CXC-motif-chemokine-ligand-8 (CXCL8, IL8). We further identify a so-far unknown connection between endothelial PRKD1 and LTB_4_ biosynthesis in PMNs that is mediated by altered post-traumatic sEV cargo and secretion.

## 2 Materials and methods

Key materials are listed in **Supplementary Table S1**.

### 2.1 Ethics committee approval

Local ethics committee approval: 116/14 & 261/22 for peripheral blood from healthy probands, 94/14 for peripheral blood from polytrauma patients. Anonymized information on patient and proband data is published in Seibold et al., 2021 (14).

### 2.2 Thorax trauma, polytrauma as well as polytrauma with hemorrhagic shock experiments in mice

All mouse experiments were performed in adherence with the National Institute of Health Guidelines on the Use of Laboratory Animals and the European Union “Directive 2010/63/EU on the protection of animals used for scientific purposes” (animal experiment approval numbers: 1413, 1258). Immunohistochemistry staining for PRKD activity in mouse lungs was performed on samples from polytrauma as well as polytrauma with hemorrhagic shock mice acquired from a previous study described in Denk et al., 2018 (15). In brief, to mimic polytrauma in patients, anesthetized mice received a blunt chest trauma, head injury, femur fracture, and soft tissue injury. Additional hemorrhagic shock was induced by drawing blood to reach a mean arterial blood pressure of 30 mm Hg for 60 min upon which mice were resuscitated with a balanced electrolyte solution for 30 min. All mice were sacrificed 4 h post injury and organs as well as blood were harvested. Lungs were embedded in paraffin for further analysis (15). We have subsequently conducted immunofluorescence staining’s on embedded FFPE samples of lung tissue sections from 4 mice each with polytrauma or polytrauma with hemorrhagic shock as well as the respective sham controls as described in 2.5.

TxT experiments were conducted by inducing a defined bilateral lung contusion using an air pressure blast wave on anesthetized 12-week old male C57BL/6 animals (14). The level of pulmonary contusion was chosen based on histologic, cardiopulmonary as well as immunologic changes in earlier studies and was sufficient to induce a profound local and systemic inflammatory response, but without being lethal itself (16). Mice were sacrificed 4 h after the traumatic insult and blood plasma as well as tissue samples were frozen at -80°C for further analyses. The respective mouse experiments were conducted as part of a previous study published in Seibold et al., 2021 (14). Lysates of the lung tissue for Western blots were generated as described in 2.4.

### 2.3 Cell culture

Human umbilical vein cells (HUVECs, ATCC, CRL-1730) were maintained in endothelial cell growth medium MV2 (PromoCell) with MV2 supplement with 1% penicillin and streptomycin. HEK293T cells (ATCC) were maintained in DMEM with 10% fetal calf serum supplemented and 1% penicillin and streptomycin. All cells were kept at 37°C in 5% CO2. To mimic a traumatic microenvironment *in vitro*, we stimulated HUVECs with a polytrauma cocktail (PTC) of cytokines, chemokines and anaphylatoxins, consisting of Intereukin1-beta, Interleukin 6, CXC-motif-chemokine-ligand-8, complement component-3a, complement component 5a-des-Arg at concentrations measured in patient serum 24 h after polytrauma (17) (IL1β, 200 pg/mL; IL6, 500 pg/mL; CXCL8, 150 pg/mL; C3a, 500 ng/mL; C5a-des-Arg, 10 ng/mL). The role of PRKD in post-traumatic hyperinflammation is investigated in a number of experiments by employing the specific PRKD small molecule inhibitors CRT0066101 and Kb-NB-142-70 (9, 18). Both inhibitors work at nanomolar concentrations. For CRT0066101, IC50 values for PRKD1, 2, 3 are reported as 1, 2.5, or 2 nM, respectively (19). Kb-NB-142-70 inhibits PRKD1, 2, 3 with an IC50 of 28.3, 58.7 and 53.2 nM (20). In cell culture experiments both inhibitors were applied at a concentration 5 µM.

### 2.4 Preparation of tissue lysates from mice

Organs were cut up into smaller pieces and immediately transferred into cryo-tubes that were subsequently stored on dry ice. For long-term storage the tubes were transferred to the freezer set to -80°C on the same day. Tissue lysates were prepared by addition of NP-40 Lysis buffer [150 mM sodium chloride, 1% NP-40, 50 mM Tris pH 8.0] (14) supplemented with 1 × Protease Inhibitor Cocktail and 1× Phosphatase Inhibitor Cocktail (both Sigma–Aldrich) to 20 mg of frozen tissue pieces in a 2 mL reaction tube. Homogenization of samples was achieved using a TissueLyser (Qiagen) at a frequency of 50 s^–1^ for 5 min. The samples were centrifuged for 20 min at 12000 rpm at 4°C and the cleared lysate was stored at -20°C.

### 2.5 Immunofluorescence staining on lung section and quantitative confocal microscopy

Immunofluorescence (IF) staining on lung tissue sections from mice was conducted as described previously (13, 21). The lung tissue sections (5µm thickness) were deparaffinized and rehydrated using xylene and a graded alcohol series. The masked epitope was revealed by heat-induced epitope retrieval using Dako antigen retrieval solution (Dako #S1699). Subsequently, the mix of primary antibodies CD34 (#ab8158 Abcam; dilution 1:100) and Phospho-PKD/PKCμ (Ser916) (#2051 Cell Signaling; dilution 1:100) diluted in TBS + 0,05% Tween was applied on tissue sections and incubated under humidified atmosphere overnight at 4°C. Following washing with TBS-Tween, tissue sections were incubated for one hour at RT with the combination of fluorophore labelled secondary antibodies (goat anti-Rabbit IgG (H+L)-Alexa Fluor™ 647, #A21244; goat anti-Rat IgG (H+L)-Alexa Fluor™ 568 ‚#A110077). Sections were mounted using ProLong™ Gold Antifade mount. Nuclei were stained with 4′,6-Diamidin-2-phenylindol (DAPI). Images were acquired by a Leica TCS SP8 confocal laser scanning microscope in sequential scan mode with equal settings using a HC PL APO CS2 40x/1.30 OIL objective and HyD detectors. pPRKD^S916^ signal intensities (8-bit) in airy confocal sections were quantified by generating detection masks from the CD34 signal using NIH ImageJ with the following commands: Threshold, close, erode, analyze particles (10-infinity; show masks; add to manager; include holes; summary).

### 2.6 RNA isolation

Cells from cell culture experiments were lysed directly in the well by addition of 700 µL QIAzol lysis reagent (Qiagen). The wells were incubated for 5 min. Next, the contents of the wells were transferred to a new tube and RNA was isolated with the miRNeasy^®^ Mini Kit (Qiagen) according to the manufacturer’s instructions.

### 2.7 Quantitative real-time PCR

RNA was extracted from cells with the miRNeasy^®^ Mini Kit (Qiagen) according to the manufacturer’s instructions. cDNA was prepared from 200 ng of total RNA, using the iScript™ cDNA Synthesis Kit (Biorad) for mRNA. qPCR reactions were performed with PowerUpTM SYBRTM Green Master Mix (Applied Biosystems) (14). The thermal cycling conditions were set as follows: 2 min at 95°C, followed by 45 cycles of 15 s at 95°C denaturation and 1 min at 60°C for the anneal/extension phase. The relative gene expression levels were calculated by the “delta-delta Ct-method”.

### 2.8 sEV Isolation from plasma

Blood samples were subjected to centrifugation at 2000 rpm for 10 min at RT to separate the plasma from the cellular fraction. Hereafter, the samples were centrifuged at 13000 rpm for 20 min and the resulting plasma supernatants were transferred to new LoBind reaction tubes (Eppendorf). The sEVs were purified from the plasma by size exclusion chromatography with Exo-Spin™ columns (Cell Guidance Systems) according to the manufacturer’s instructions as described previously (13, 14).

### 2.9 sEV isolation from cell culture supernatants

HUVECs were grown to a confluency of ~ 90%, cells were washed with PBS and incubated in fresh serum-free MV2 medium for a duration of 16 h at 37°C, 5% CO_2_. The supernatants were harvested, processed by centrifugation and 0.2 µm filtration to remove debris, concentrated using ultrafiltration at 2000 rpm, 4°C by Vivaspin Turbo 15 (100 000 MWCO) units (Sartorius, #VS15T41) followed by precipitation and size exclusion chromatography with the Exo-Spin™ system (Cell Guidance Systems) according to the manufacturer’s instructions and as described previously in Seibold et al., 2021 (14).

### 2.10 Coimmunoprecipitation experiments

Coimmunoprecipitation experiments (CoIPs) were performed as described previously (21) by precipitating VE-cadherin from HUVEC cell lysates and detection of coprecipitated binding partners Cortactin, Vinculin and β-catenin in the immunoprecipitated samples from shScramble, shPRKD1 stable HUVEC knockdown cell lines as well as cells treated with the PRKD inhibitor CRT0066101 (5 µM). Samples were analyzed by Western blot.

### 2.11 Cell lysates and Western blot

To generate total cell lysates, cells were treated with lysis buffer (50 mM Tris, pH 7.4, 150 mM NaCl, 5 mM MgCl_2_, 1% Triton X-100, supplemented with 1 × Protease Inhibitor Cocktail and 1× Phosphatase Inhibitor Cocktail. The resulting supernatants were mixed with 5x Laemmli Sample Buffer and incubated at 95°C for 10 min. The samples were then subjected to SDS-PAGE followed by Western blotting and detection by ECL as described previously (14).

### 2.12 Nanoparticle-tracking analysis of sEVs

NTA was performed using a Nanosight NS300 device with the respective software (Malvern Panalytical). Isolated sEVs were diluted in particle-free sterile filtrated PBS and injected using a syringe pump. Particles were tracked under constant flow. Each sEV sample was measured three times for 60 s.

### 2.13 Transmission electron microscopy of sEVs

The purified sEVs were subjected to negative staining. To this end, 5 µL of each sample was added onto a glow discharged carbon-coated copper TEM grid and incubated for 1 min. Next, the grid was washed 3 x with 7 µL of Mili-Q water and stained 3 x with 7 µL of 3% uranyl acetate. During the last staining step, the uranyl acetate drop remained 30 s on the grid before the liquid was removed and the grid was left to dry for at least 15 min. The grids were imaged using a JEOL 1400 TEM.

### 2.14 Isolation of human PMNs

Healthy donors were subjected to venipuncture and blood samples were collected in EDTA monovettes. The isolation of PMNs was conducted using Polymorphprep (Axis Shield) according to the manufacturer’s instructions. In brief, 5 mL of blood where overlayed on top of the same volume Polymorphprep in a 15 mL tube and centrifuged at 550 x g for 35 min at RT. The resulting layers containing the PMNs were pooled and mixed with RPMI media in a 1:10 ratio. The cells were pelleted by centrifugation at 400 x g for 10 min and afterwards the supernatant was discarded and the pellet was resuspended in RPMI media (9).

### 2.15 PMN transmigration assay

A number of 8x10^4^ HUVEC cells per insert were seeded onto Corning Costar Transwell filters with 5 μm pore size (Sigma-Aldrich). The cells were grown for 48 h at which point PTC, CRT0066101 (5 µM) or DMSO were added to the upper compartment of the filters and incubated for the indicated time periods. Then, the medium with the inhibitors was removed and 100 µl of fresh MV2 medium was added to the upper compartment of the filter. The filter inserts were transferred to a new plate with 800 µl of MV2 medium per well containing chemoattractant (fMLP, 100 nM) or DMSO.

Human PMNs were harvested as described in 2.14 and labeled with CellTracker Deep Red Dye (Invitrogen) for 1 h as stated by the manufacturer. Finally, a number of 5x10^5^ labelled PMNs was added to the upper compartment of the inserts to initiate the experiment. After 1 h of transmigration the filter inserts were removed and relative fluorescence intensities in the respective wells was measured using a Tecan M200Pro plate reader in scanning mode (excitation: λ = 630 nm, emission: λ = 660 nm).

### 2.16 PMN adhesion assay

A number of 2x10^4^ HUVEC cells per well were seeded in a 96-well plate and grown for 48 h. The supernatants of the wells were discarded and PTC, CRT0066101 (5 µM) or DMSO in MV2 medium was added to the appropriate wells and incubated for 4 h at which point the supernatant was removed. Human PMNs were harvested and labelled with CellTracker Deep Red as described 2.15. The labelled PMNs were added to the wells (1x10^5^ PMNs/well) and incubated for 1 h at 37°C. To remove non-adhesive PMNs, the plate was washed three times with RPMI and analyzed by measurement of relative fluorescence intensities using a Tecan M200Pro plate reader in scanning mode (excitation: λ = 630 nm, emission: λ = 660 nm).

### 2.17 Transendothelial electrical resistance

A number of 8x10^4^ HUVEC cells per insert were seeded onto Corning Costar Transwell filter inserts with 5 μm pore size (Sigma Aldrich) and grown for 48 h. The compartment in the top of the insert was filled with 200 µL of MV2 medium containing Thrombin, PTC, CRT0066101 (5 µM) or DMSO and filters were transferred into a CellZscope TEER device (NanoAnalytics) where TEER was measured by impedance spectroscopy (14).

### 2.18 Transendothelial flux assay with FITC-albumin

A number of 1.2x10^5^ HUVEC cells per insert were seeded onto Corning Costar Transwell filter inserts with 5 μm pore size (Sigma Aldrich) and grown for 48 h. At this point DMSO, Thrombin, PTC, CRT0066101 or Kb-NB-142-70 (both 5 µM) were added to the upper compartment of the filters and incubated as indicated. These solutions also contained FITC-albumin (Sigma–Aldrich) in a concentration of 40 µg/mL. After the incubation period, the filter inserts were removed and the FITC fluorescence was measured in the bottom well using a Tecan M200Pro plate reader (excitation: λ = 490 nm, emission: λ = 520 nm).

### 2.19 Enzyme-linked immunosorbent assays

All ELISAs were performed according to the manufacturer’s instructions. For IL6 (Thermo Scientific), CXCL8 (R&D systems) and Leukotriene B_4_ (Enzo, R&D systems) cell culture supernatants from HUVECs or shPRKD1-knockdown HUVEC cell lines were used. Standard curves were created using non-linear- 4P- regression.

### 2.20 Flow cytometry experiments

HUVEC cells grown in 6-well plates and treated with DMSO, PTC, CRT0066101 (5 µM) or Kb-NB-142-70 (5 µM) as indicated. Cells were washed with PBS and detached by PBS supplemented with EDTA (Sigma–Aldrich) for 3 min. The cells were resuspended in FACS buffer (PBS, 5% FCS, 0.05% EDTA) that contained the fluorescently labelled primary-antibodies (Miltenyi Biotec). Staining was performed on ice for 1 h. Flow cytometry was done using an LSRII special order FACS System (BD Bioscience) or an Attune NxT analytical flow cytometer (Thermo Scientific). Data was evaluated and plotted with Flowjo (Agilent).

### 2.21 Generation of stable shRNA-kockdown cell lines

Lentiviruses were produced by transfecting HEK293T cells at 80% confluency using Lipofectamine 3000 (Invitrogen) with psPAX2 and pMD2.G packaging constructs as well as the respective shRNAs (**Supplemental Table S1**) as described previously (22, 23). The supernatants containing the virus were harvested after 48 h and stored at -80°C. HUVEC cells were transduced with the respective lentivirus and subsequently selected with puromycin (6 µg/mL) for 6 weeks to generate stable shRNA cell lines.

### 2.22 Re-expression of PRKD1 in HUVEC shRNA-knockdown cells

For nucleofection of pcDNA3-PRKD1 (24) expression constructs, the HUVEC nucleofector kit (Lonza) was used according to the manufacturer’s instructions and as described previously (14).

### 2.23 Mass spectrometry

Mass spectrometry was performed on sEVs from DMSO and CRT0066101 (5 µM) treated HUVECs that were isolated after 16 h of treatment (n = 5 samples per condition). The sample preparation and analysis were performed as described in Seibold et al., 2021 (14).

### 2.24 Knockdown experiments with siRNAs

A number of 1x10^5^ HUVEC cells were seeded in a 24-well plate and transfected at 90% confluency with the indicated siRNAs using Lipofectamine 3000. After 48 h, the supernatants were discarded and the cells were treated with PTC, CRT0066101 (5 µM) or DMSO.

### 2.25 Coculture cultivation for LTB_4_ synthesis and respiratory burst

HUVEC cells were seeded into a 24-well plate at a concentration of 1x10^5^ cells/well. After 24 h, the supernatants were discarded and 5x10^5^ human PMNs were added, followed by PTC, CRT0066101 (5 µM) or DMSO treatment. This coculture was incubated for 3 h 45 min at which point fMLP was added (100 nM). After 15 min the supernatants were collected, centrifuged for 2 min at 2000 rpm at RT and stored at -20°C. Respiratory burst experiments with PMNs were performed using the Neutrophil/Monocyte Respiratory burst assay kit (#601130, Cayman Chemical) according to the manufacturer´s instructions. PMNs were incubated with DHR 123 reagent at a concentration of 1x10^5^ cells/mL in Assay Buffer (RPMI, 1 mM Calcium Chloride) for 30 min before they were added to HUVECs that had been seeded into a 24-well plate at a concentration of 1x10^5^ cells/well and grown for 24 hours. This coculture was incubated for 3 h 45 min at which time point the PMNs were harvested. Next, fMLP was added (100 nM) to the harvested PMNs and incubated for 15 min. Subsequently, PMNs were resuspended in FACS buffer, stored on ice and analyzed by flow cytometry using the FITC-A channel.

### 2.26 Supernatant treatment experiments for the generation of LTB_4_ and respiratory burst

HUVEC cells were seeded into a 24-well plate and incubated until 90% confluency had been reached. At this point, DMSO, PTC, CRT0066101 (5 µM) or Kb-NB-142-70 (5 µM) were added to the respective wells and incubated for 4 h, as indicated. These supernatants were then harvested and added to PMNs that were purified from whole blood. This mixture was incubated again for 4 h, then fMLP was added at a concentration of 100 nM. After 15 min the supernatants were collected by centrifugation for 2 min at 2000 rpm at RT and stored at -20°C. For the respiratory burst assays, the respective supernatants were added to PMNs pretreated with DHR 123 reagent at a concentration of 1x105 cells/mL in Assay Buffer (RPMI, 1 mM Calcium Chloride) for 30 min. PMNs were incubated for 3 h 45 min at which time point fMLP was added at a concentration of 100 nM. After 15 min the PMNs were collected by centrifugation, resuspended in FACS buffer, stored on ice and analyzed by flow cytometry using the FITC-A channel.

### 2.27 Immunofluorescence and quantitative analysis

For confocal imaging, immunofluorescence staining was performed as described previously (25, 26). In brief, 1.5 × 10^5^ HUVEC cells were seeded on coverslips in 12-well plates and grown to confluency. Subsequently, HUVEC monolayers were treated with PTC, CRT0066101 (5 µM) or DMSO for 8 h. Samples were fixed using formaldehyde (3.75%) for 20 min at RT and permeabilized with Triton X-100 (0.1%) in PBS for 30 s. After blocking (5% FCS, 0.05% Tween-20 in PBS) for 20 min, samples were stained with primary antibodies against VE-cadherin, β-catenin overnight at 4°C. Samples were washed three times with PBS and incubated with secondary antibodies conjugated with the respective Alexa fluorophores as well as Phalloidin-Alexa-Fluor-647 (1:60) and DAPI (Thermo Scientific) for 1 h at RT. Coverslips were mounted in Fluoromount-G (Thermo Scientific). Intensity quantification was performed as described previously using NIH ImageJ (26). Experiments were analyzed by a confocal laser scanning microscope TCS-SP8-HCS (Leica) equipped with an HC PL APO CS2 40×/1.3 oil immersion objective. Images were acquired in sequential scan mode with equal settings within the linear range of detectors. Quantitative mean of ROI analysis was conducted by NIH ImageJ, averaging intensities from 3 similar-sized sub-ROIs per junction.

### 2.28 Enrichment analysis using EnrichR

Unweighted enrichment analysis for MS data was performed using the EnrichR meta-analysis web tool (https://amp.pharm.mssm.edu/Enrichr/) to query the GO Biological process database with up- or down-regulated proteins (cutoff of (log 2≥ +1,5, ≤-1,5)).

### 2.29 Medical art illustrations

Illustrations were created using Servier medical art templates (https://smart.servier.com/) with minor modifications according to terms of the creative commons attribution 3.0 license agreement (https://creativecommons.org/licenses/by/3.0/).

### 2.30 Quantification and statistical analysis

Quantification of Western blot bands was conducted using ImageJ “Gels Submenu” or “Gel plotting macros” (https://imagej.nih.gov/nih-image/manual/tech.html#analyze). Statistical analysis was performed using Prism software, version 9.4 (GraphPad, San Diego, CA). Graphs depict mean ± SEM for all conditions. Statistical significance: ns, not significant, *P = 0.05–0.01, **P = 0.01–0.001, ***P < 0.001, ****P < 0.0001.

## 3 Results

### 3.1 PRKD is activated in endothelial cells by post-traumatic cytokines *in vitro* and *in vivo*

**Figure 1:**
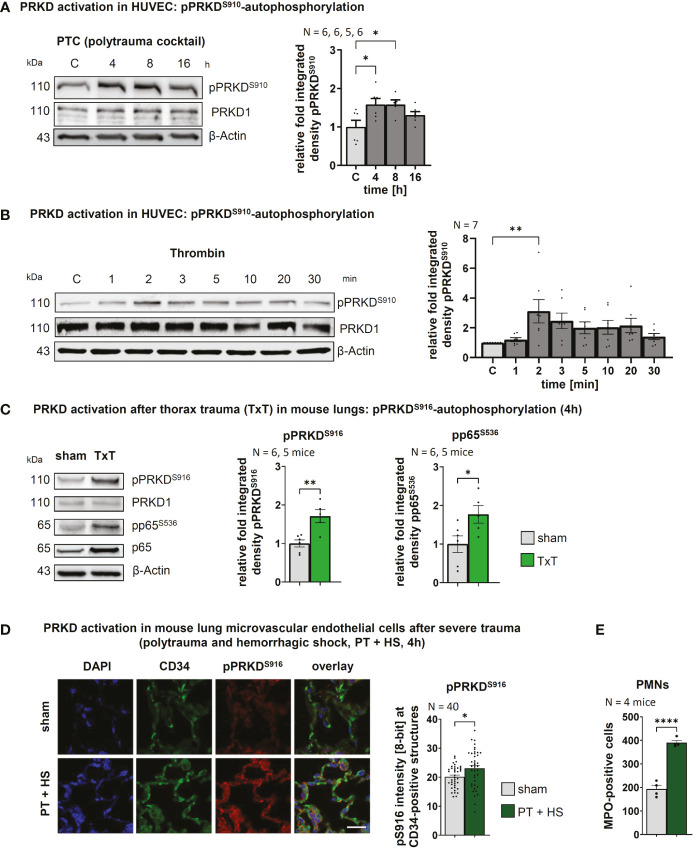
PRKD is activated in endothelial cells after *in vitro* & *in vivo* trauma **(A)** PRKD activation kinetic in HUVEC cells detected by pPRKD^S910^-autophosphorylation after treatment with polytrauma cocktail (PTC: IL1β, IL6, CXCL8, C3a and C5a-des-Arg). The graph shows the quantitative analysis of Western blots by measuring integrated density normalized to β-Actin. **(B)** PRKD activation kinetic in HUVEC cells detected by pPRKD^S910^-autophosphorylation after treatment with thrombin (5U). The graph shows the statistical analysis of Western blots quantified as indicated in **(A)**. **(C)** PRKD activation in mouse lungs 4h after thorax trauma (TxT), as detected by pPRKD^S916^-autophosphorylation. Pro-inflammatory signaling was measured using pP65^S536^-phosphorylation in Western blots. **(D)** PRKD activation detected by pPRKD^S916^-autophosphorylation in lung microvascular endothelial cells of mice (N = 4 mice/10 images per immunohistochemistry staining section) after polytrauma with hemorrhagic shock (PT + H S, 4 h). PRKD activation was detected by pPRKD^S916^-immunofluorescence of lung sections costained with the endothelial marker CD34. Images from airy confocal sections were acquired using a confocal microscope with equal settings. Scale: 20 µm. Quantitative mean of ROI analysis for endothelial cells was performed by generating detection masks using the CD34 signal in NIH ImageJ. **(E)** Infiltration of PMNs into lungs of polytrauma mice with hemorrhagic shock quantified by immunofluorescence for MPO-positive cells in lung sections. Unless stated otherwise, N-numbers indicate the number of independent samples. Statistical tests: **(A, B)** One-way ANOVA with Dunnett’s multiple comparison post-test; **(C, D, E)** Two-tailed unpaired student’s t-test *P < 0.05; **P < 0.01; ****P < 0.0001; ns: no significant difference.

The scope of the post-traumatic immune response is critically determined by the coordinated interaction of PMNs with the endothelium that is controlled at multiple levels in both cell types. We have recently shown that PRKD1 is implicated in modulating neutrophil cell motility upon trauma (9). Since PRKD is activated by cellular stresses, e.g., reactive oxygen species (27) and also modulates cell-cell adhesion (21), we were prompted to investigate, the role of PRKD in the context of the post-traumatic innate immune response of the endothelium. To this end, we stimulated human umbilical vein cells (HUVECs) *in vitro* with a polytrauma cocktail (PTC), consisting of Intereukin1-beta, Interleukin 6, CXC-motif-chemokine-ligand-8, complement component-3a, complement component 5a-des-Arg (IL1β, IL6, CXCL8, C3a and C5a-des-Arg) at concentrations measured in patient serum 24 h after polytrauma (17). Indeed, PRKD was activated and stayed active between 4 to 8 h after stimulation, as measured by pPRKD^S910^-autophosphorylation of the kinase (26). After 16 h PRKD activity returned to almost basal levels **(** **Figure 1A** **)**. PRKD activation at the 4 h time point correlates with a peak for cytokine transcripts in lungs after severe blunt chest trauma (14, 28). PRKD was also activated by the barrier-destabilizing agent thrombin (29). Compared to PTC, activation of PRKD by thrombin was fast, peaking 2 min after stimulation followed by a drop to almost basal levels within 30 min **(** **Figure 1B** **)**. Excessive non–wound-related thrombin generation (30, 31) is a hallmark of acute traumatic coagulopathy (ATC) and associated with poor outcome (31). It is caused by a combination of tissue trauma with second hits, such as shock, hemodilution, hypothermia or acidosis (31). Thus, PRKD is activated by important trauma-associated stimuli *in vitro* with distinct kinetics. To examine activation *in vivo*, we used a well-established trauma model and measured PRKD activity and concomitant nuclear factor k-light-chain-enhancer of activated B cells (NFκB) activation by pP65^S536^-phosphorylation to indicate pro-inflammatory signaling in lung lysates 4 h after blunt chest trauma (TxT) in mice (14). **Figure 1C** shows both, significantly increased pPRKD^S910^- as well as NFκB pP65^S536^- phosphorylation after TxT compared to sham controls. *In vivo* activation of PRKD in endothelial cells upon trauma was examined by immunohistochemistry in lung samples of mice that were previously subjected to severe polytrauma with and w/o hemorrhagic shock (PT +/- HS) to elicit a major trauma response (15). Polytrauma in combination with hemorrhagic shock is known to increase overall mortality in patients, whereby hemorrhagic shock significantly contributes to the systemic release of cytokines, such as IL6. In these experiments, increased costaining of pPRKD^S916^-autophosphorylation with the endothelial marker CD34 was detected by confocal imaging concomitant with infiltration of PMNs in lung sections **(** **Figures 1D, E** **)**. Also, polytrauma without hemorrhagic shock resulted in a similar trend **(** **Figure S1A** **)**. Thus, our data demonstrate, PRKD is activated in endothelial cells upon trauma *in vitro* and *in vivo* **(** **Figures 1A–D** **)**.

### 3.2 Protein kinase D1 activity determines the stability of the endothelial barrier *in vitro*

**Figure 2:**
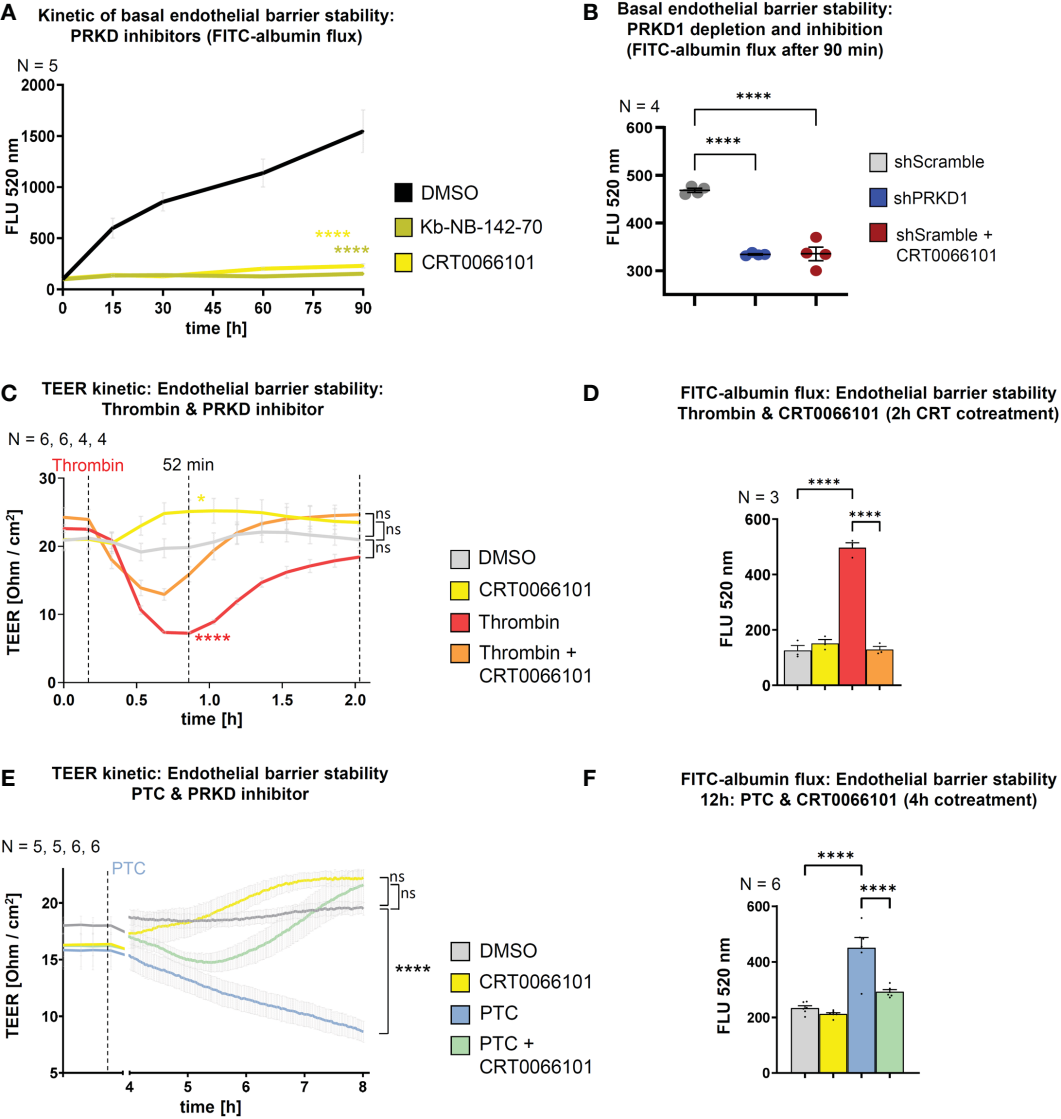
PRKD modulates endothelial barrier stability. **(A)** Kinetic of basal endothelial barrier stability for HUVEC cells upon incubation with PRKD inhibitors Kb-NB-142-70 or CRT0066101 (both 5 µM) measured by FITC-albumin flux for 90 min. **(B)** Endothelial barrier stability of HUVECs under basal conditions upon stable knockdown of PRKD1 by shRNA or CRT0066101 treatment measured by FITC-albumin flux across confluent, differentiated HUVEC monolayers after 90 min. **(C)** Kinetic measurement of endothelial barrier stability for HUVECs treated with thrombin and CRT0066101 quantified by trans-endothelial electrical resistance (TEER) for 2 h. **(D)** Endothelial barrier stability of HUVECs treated with thrombin and CRT0066101 measured by FITC-albumin flux after 2 h as indicated in **(B)**. **(E)** Kinetic measurement of endothelial barrier stability for HUVECs treated with PTC and CRT0066101 quantified by TEER for 8 h. **(F)** Endothelial barrier stability of HUVECs treated with PTC and CRT0066101 measured by FITC-albumin flux after 12 h with CRT0066101 coincubation for the final 4 h. N-numbers indicate the number of independent samples. Statistical tests: **(A–F)** One-way ANOVA with Tukey’s multiple comparison post-test. ****P < 0.0001; ns: no significant difference.

Endothelial barrier stability is a critical pathophysiological parameter determining the passage of PMNs towards the site of injury (32, 33). Barrier integrity requires VE-cadherin adhesion contacts at the plasma membrane (34) and since PRKD1 has been implicated in the regulation of E-cadherin mediated cell-cell adhesion (21), we went on to characterize the functional state of the barrier using FITC-albumin flux experiments and transendothelial electrical resistance measurements (TEER) across differentiated HUVEC monolayers on transwell filters (14). A potential role of PRKD activity was examined using the selective PRKD small molecule inhibitors CRT0066101 and Kb-NB-142-70 (9, 18). Both inhibitors have been described to work at nanomolar concentrations. For CRT0066101, IC50 values for PRKD1, 2, 3 were determined at 1, 2.5, or 2 nM (19). The Kb-NB-142-70 inhibitor was shown to inhibit PRKD1 with an IC50 of 28.3 ± 2.3 nM, PRKD2 with 58.7 ± 4.2 and PRKD3 at 53.2 ± 3.5 nM (20). Thus, we have applied both inhibitors at 5 µM concentrations to evaluate if endothelial barrier stability is affected by inhibition of PRKD kinase activity. Interestingly, both inhibitors strongly increased basal endothelial barrier stability in FITC-albumin flux experiments across Transwell filters **(** **Figure 2A** **)**. A similar observation was made by shRNA mediated knockdown of the PRKD1 isoform (shPRKD1), whereas shScramble was used as a control, suggesting that depletion of PRKD1 is indeed sufficient to stabilize barrier integrity **(** **Figure 2B** **)**. Knockdown of PRKD1 by shRNA in stable cell lines was confirmed by Western blots (**Figure S1H** **)**. Of note, at the CRT0066101 concentrations of 5 µM used in assays, overall cell viability and numbers of HUVEC cells were only mildly affected after 8 h **(** **Figure S1B** **)**. To assess whether PRKD inhibition would revert destabilization of the endothelial barrier by thrombin or PTC, we performed additional TEER and FITC-albumin flux experiments. In line with Seibold et al., 2021 (14), destabilization of the endothelial barrier by thrombin was fast and peaked around 35 min after thrombin stimulation in TEER assays (T52 min). Inhibition of PRKD by CRT0066101 enhanced basal barrier stability and reverted barrier destabilization by thrombin **(** **Figure 2C** **)**. FITC-albumin flux assays performed 2 h after thrombin stimulation verified the marked destabilization of the endothelial barrier by thrombin that was fully recovered upon cotreatment with CRT0066101 **(** **Figure 2D** **)**. TEER measurement upon incubation of HUVEC cells with PTC revealed a slow, gradual decline in barrier stability that continued until the end of the measurement period at 8 h, where significant barrier destabilization was observed. Again, the barrier was stabilized when cells were incubated with PTC and CRT0066101 in combination as compared to PTC, on its own **(** **Figure 2E** **)**. FITC-albumin flux also demonstrated a significant restabilization of the endothelial barrier upon CRT0066101 treatment of PTC samples after 4 h, corroborating the TEER data **(** **Figure 2F** **)**. Thus, inhibition of PRKD during thrombin or PTC-mediated barrier destabilization was able to completely restore the endothelial barrier *in vitro*. The effect of PRKD inhibition on endothelial barrier stability was substantial and comparable to inhibition of Rho-associated protein kinase (ROCK), a major kinase regulator of barrier stability, when using the Y27632 inhibitor (10 µM) (35) **(** **Figure S1C** **)**.

### 3.3 PRKD activation disrupts the endothelial barrier by destabilizing VE-cadherin adhesion complexes

**Figure 3:**
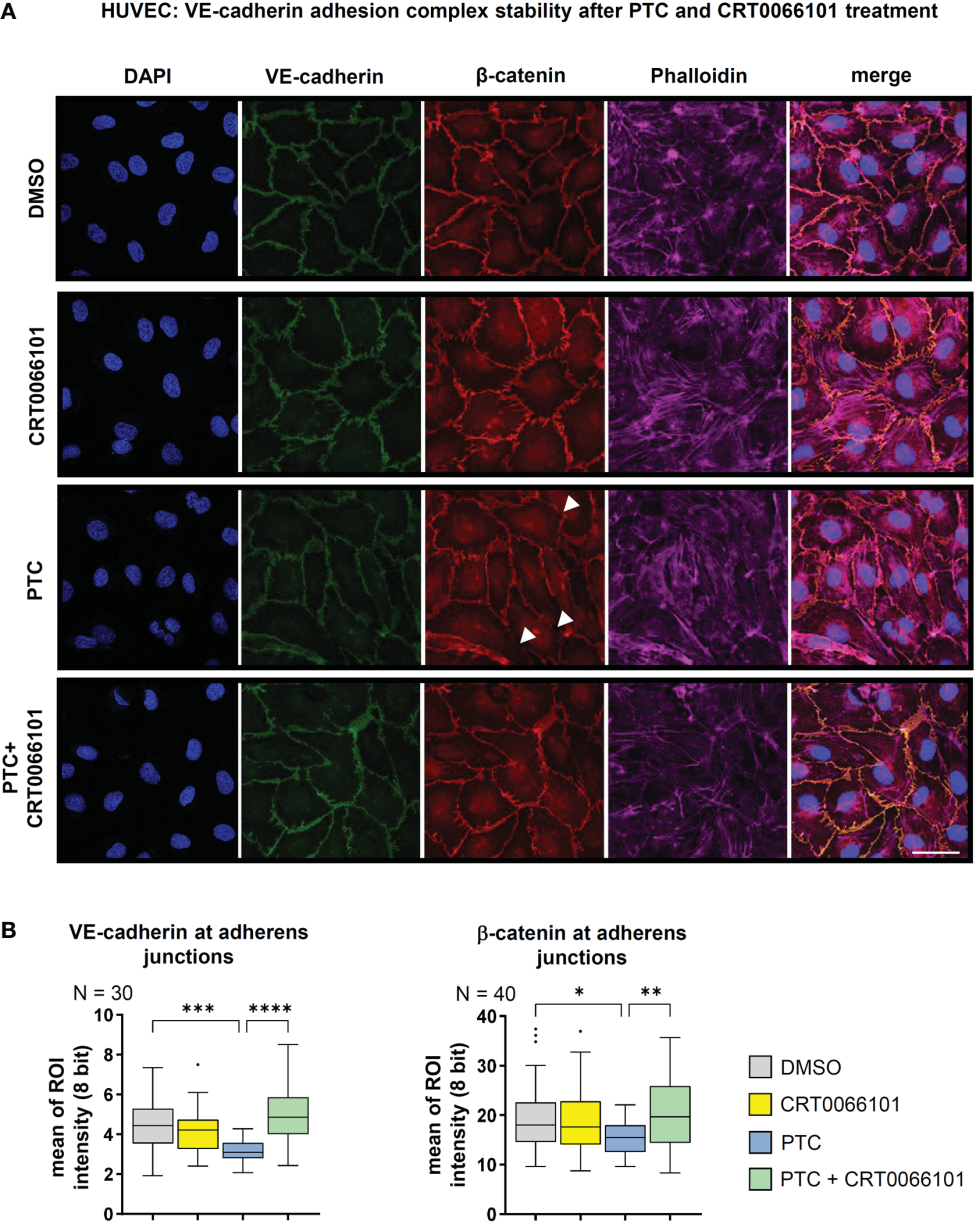
Stability of VE-cadherin adhesion complexes. **(A)** Immunofluorescence staining of confluent HUVEC cells treated with PTC and CRT0066101 for 8 h. Functional adherens junctions were detected by quantifying VE-cadherin and β-catenin intensities. F-actin was detected using Phalloidin-Alexa-647, nuclei were stained with DAPI. Scale: 50 µm. Mean of ROI analysis was performed by averaging intensities from 3 sub-ROIs per junction for N=3 images (VE-cadherin) and N=40 images (β-catenin). **(B)** Quantification of VE-cadherin and β-catenin intensities at adherens junctions in immunofluorescence stainings. N-numbers indicate the number of quantified junctions (1 junction per image, 3 independent stainings). Statistical tests: **(A)** One-way ANOVA with Tukey’s multiple comparison post-test. *P < 0.05; **P < 0.01; ***P < 0.001, ****P < 0.0001; ns: no significant difference.

**Figure 4:**
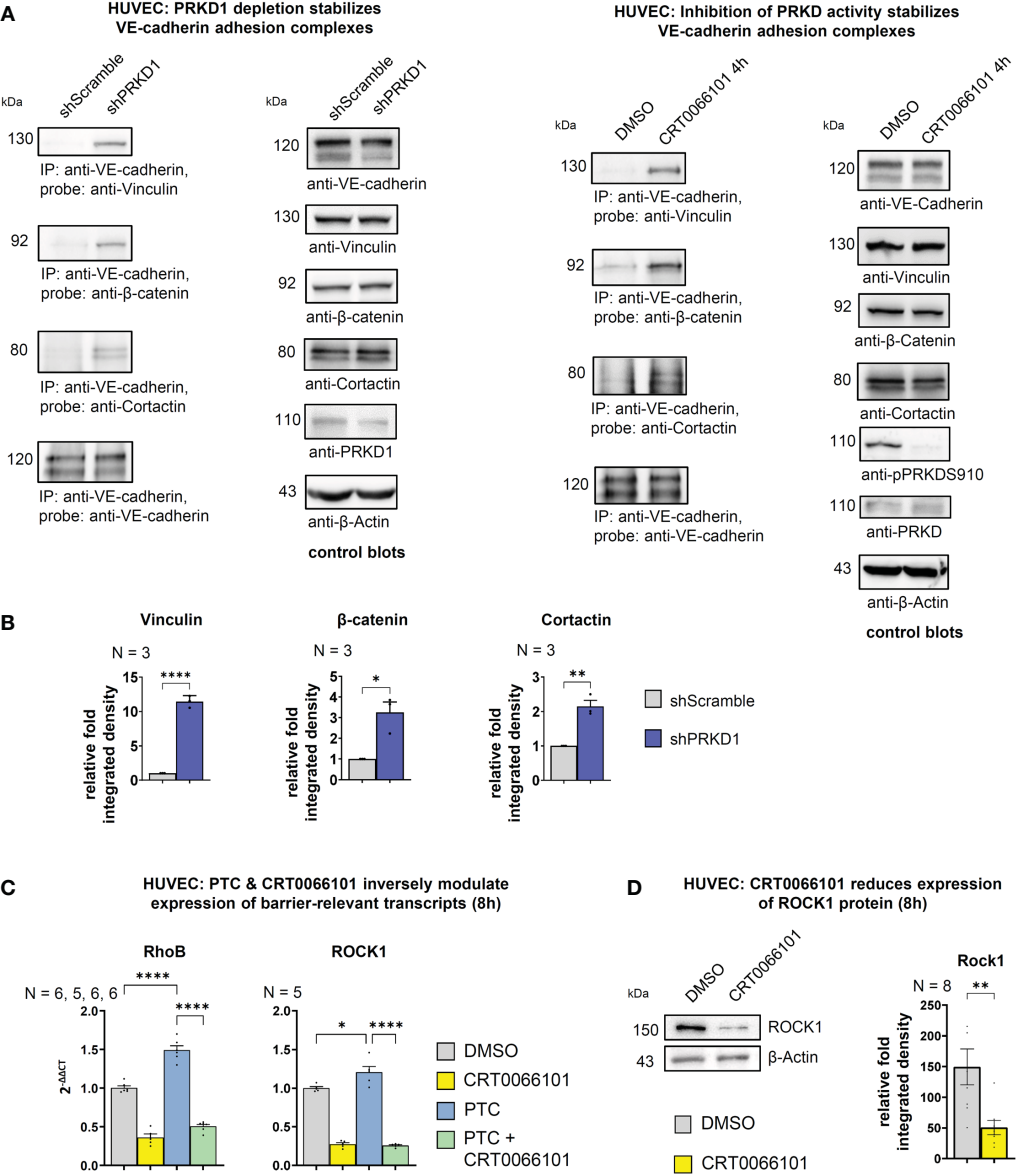
Molecular composition of VE-cadherin adhesion complexes **(A)** Immunoprecipitation of VE-cadherin from stable HUVEC cell lines treated with shRNAs against PRKD1 or HUVECs treated with CRT0066101 demonstrate enhanced coprecipitation of Vinculin, β-catenin and Cortactin, when PRKD1 was depleted or inhibited. **(B)** Quantitative analysis of coprecipitated proteins as shown in **(A)** normalized to VE-cadherin. **(C)** Expression analysis of barrier destabilizing transcripts for RhoB and ROCK1 by qPCR upon PTC or CRT0066101 incubation **(D)** Expression of ROCK1 detected by Western blot normalized to β-Actin. N-numbers indicate the number of independent samples. Statistical tests: **(B, D)** Two-tailed unpaired student’s t-test; C) One-way ANOVA with Tukey’s multiple comparison post-test. *P < 0.05; **P < 0.01; ****P < 0.0001; ns: no significant difference.

Our data suggest that PRKD prominently modulates endothelial barrier stability, even under basal conditions **(** **Figures 2A, B** **)**. To uncover the underlying molecular mechanisms, we tested the expression of relevant cell adhesion and tight junction proteins upon inhibition of PRKD by CRT0066101 for 4 and 8 h, respectively. There were no changes in protein expression, even though PRKD pS910-autophosphorylation was abrogated **(** **Figure S1D** **)**. Next, we examined VE-cadherin and β-catenin levels at adherens junctions using quantitative immunofluorescence and confocal microcopy in confluent HUVEC monolayers **(** **Figure 3A, B** **)**. Upon exposure of cells to PTC for 8 h, adherens junctions appeared destabilized, as indicated by significantly lower intensities of VE-cadherin and β-catenin at the junctions. These effects were completely reversed by CRT0066101. Of note, in line with loss of adhesion complexes also junctions were physically disrupted in the PTC condition (arrow heads). Adherens junctions are stabilized by linking engaged VE-cadherin-β-catenin complexes to the underlying actin cytoskeleton (21). F-actin linkage is achieved by actin-binding proteins, such as Vinculin or Cortactin, both of which were described to further bind β-catenin (21). As we have previously shown regulation of this process for E-cadherin adhesion complexes downstream of PRKD1 phosphorylation (21), we performed coimmunoprecipitation (co-IP) experiments to investigate molecular interactions in VE-cadherin adhesion complexes upon stable knockdown of PRKD1, or inhibition of PRKD activity by CRT0066101 **(** **Figures 4A, B** **)**. In line with our previous findings (21), Vinculin, Cortactin, and β-catenin coprecipitated with VE-cadherin after knockdown of PRKD1, suggesting improved F-actin binding in the absence of PRKD. Similar effects were evident following PRKD inhibition **(** **Figure 4A** **)**. For the E-cadherin adhesion complex, we have previously demonstrated that these molecular changes are mediated by abrogating phosphorylation of the PRKD substrate Cortactin at S298 (21). In addition to PRKD, also ROCK and RhoGTPases are known regulators of adhesion complex stability (14). We thus investigated expression of the endothelial cell relevant barrier-regulatory transcripts ROCK1 and RhoB upon PTC and CRT0066101 treatment of HUVEC cells and observed a significant upregulation in PTC-treated samples (14). PRKD inhibition significantly reduced transcript levels and cotreatment of PTC & CRT0066101 significantly reversed the respective upregulation **(** **Figure 4C** **)**. To corroborate these findings, we have also shown downregulation of ROCK1 upon CRT0066101 incubation on protein level **(** **Figure 4D** **)**. Thus, our data indicate that active PRKD is involved in barrier destabilization by multiple mechanisms, including remodeling of VE-cadherin adhesion complexes and induction of the Rho-ROCK-signaling axis downstream of PTC.

### 3.4 PRKD1 activity promotes adhesion of PMNs to endothelial cells by increased surface expression of adhesion molecules downstream of NFκB

**Figure 5:**
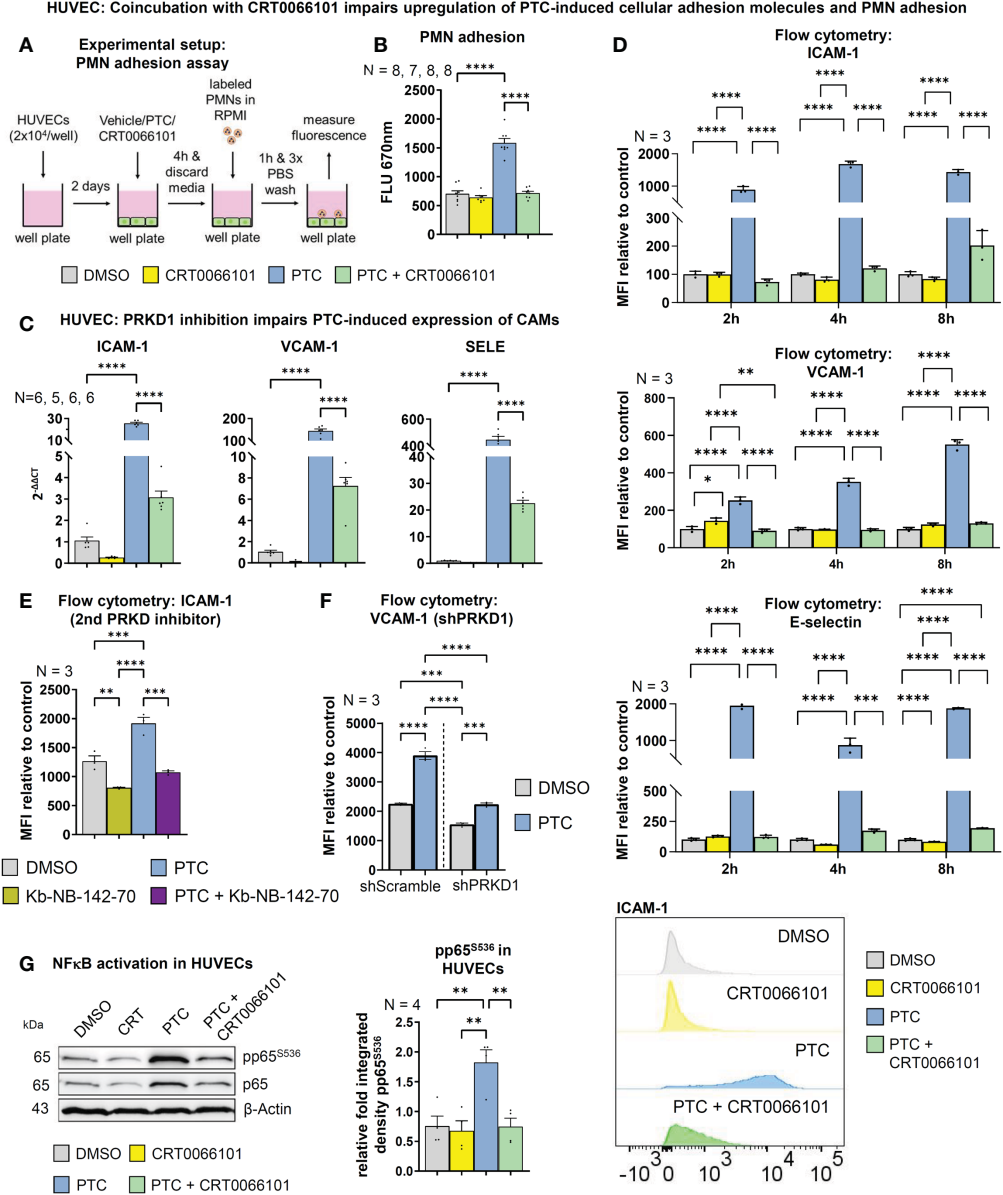
PRKD regulates expression of endothelial cellular adhesion molecules. **(A)** Experimental procedure of the adhesion assay. **(B)** For the adhesion assay HUVECs were incubated with PTC and CRT0066101 for 8 h and PMN adhesion was measured by fluorescence scanning of CellTracker Deep Red labeled PMNs using a Tecan M-200 plate reader. **(C)** Transcript levels of ICAM-1, VCAM-1 and SELE in HUVECs after incubation with PTC and CRT0066101 for 8 h, measured by qPCR. **(D)** Surface expression of ICAM-1, VCAM-1 and E-selectin measured by flow cytometry after 2, 4 and 8 h incubation. Exemplary histograms are shown for a ICAM-1 flow cytometry experiment. **(E)** Exemplary surface expression of ICAM-1 after incubation with PTC and Kb-NB-142-70 measured by flow cytometry after 8 h. **(F)** Exemplary surface expression of VCAM-1 after incubation with PTC in stable HUVEC cell lines treated with shRNAs against PRKD1 measured by flow cytometry after 8 h. **(G)** Left-hand side: NFκB activity status detected by S536-phosphorylation of the p65-subunit in HUVECs treated with PTC and CRT0066101 for 8 h. Right-hand side: Statistical analysis of the pp65^S536^-phosphorylation normalized to β-Actin. N-numbers indicate the number of independent samples. Statistical tests: **(B–G)** One-way ANOVA with Tukey’ s multiple comparison post-test. *P < 0.05; **P < 0.01; ***P < 0.001, ****P < 0.0001; ns: no significant difference.

To migrate towards an injured site, PMNs need to adhere to the endothelium, both to undergo rolling-ball migration (36), but also to initiate diapedesis (37, 38). On the endothelial side, adhesion is facilitated by the surface expression of cellular adhesion molecules, such as ICAM-1, VCAM-1 or E-selectin (10). Indeed, we found that treatment of HUVEC monolayers with PTC significantly increased PMN adhesion after 1 h. This effect was abrogated in the presence of CRT0066101, implying that PRKD activity is also involved in controlling neutrophil adhesion to the endothelium **(** **Figures 5A, B** **)**. PTC markedly increased transcript levels of cellular adhesion molecules, like ICAM-1, VCAM-1 and SELE (E-selectin) after 8 h (**Figure 5C** **)**. In line with adhesion studies, CRT0066101 treatment impaired expression of all cellular adhesion molecule transcripts and reverted PTC-mediated upregulation of cellular adhesion molecules on the RNA level. Surface expression of cellular adhesion molecules on endothelial cells was detected by flow cytometry after 2, 4 and 8 h of PTC treatment **(** **Figure 5D** **)**. Again, PTC markedly increased their respective surface presentation and the PRKD inhibitor CRT0066101 normalized expression levels. At the example of ICAM-1, this regulation was further verified using the Kb-NB-142-70 inhibitor **(** **Figure 5E** **)** as well as for VCAM-1 with knockdown of PRKD1 **(** **Figures 5F**, **S1H** **)**. Since all three cellular adhesion molecule transcripts are known NFκB target genes (39–41) and PRKDs are described to modulate NFκB activity under certain conditions (42), we determined pP65^S536^-phosphorylation as a marker for NFκB activation. In line with cellular adhesion molecule expression profiles, increased pP65^S536^-phosphorylation was detected following PTC stimulation, which was prevented by cotreatment with CRT0066101 **(** **Figure 5G** **)**. Cellular adhesion molecule expression in endothelial cells therefore is regulated downstream of PRKD1 and NFκB after stimulation with pro-inflammatory cytokines and anaphylatoxins.

### 3.5 Endothelial PRKD1 governs expression of pro-inflammatory cytokines and controls neutrophil transmigration

**Figure 6:**
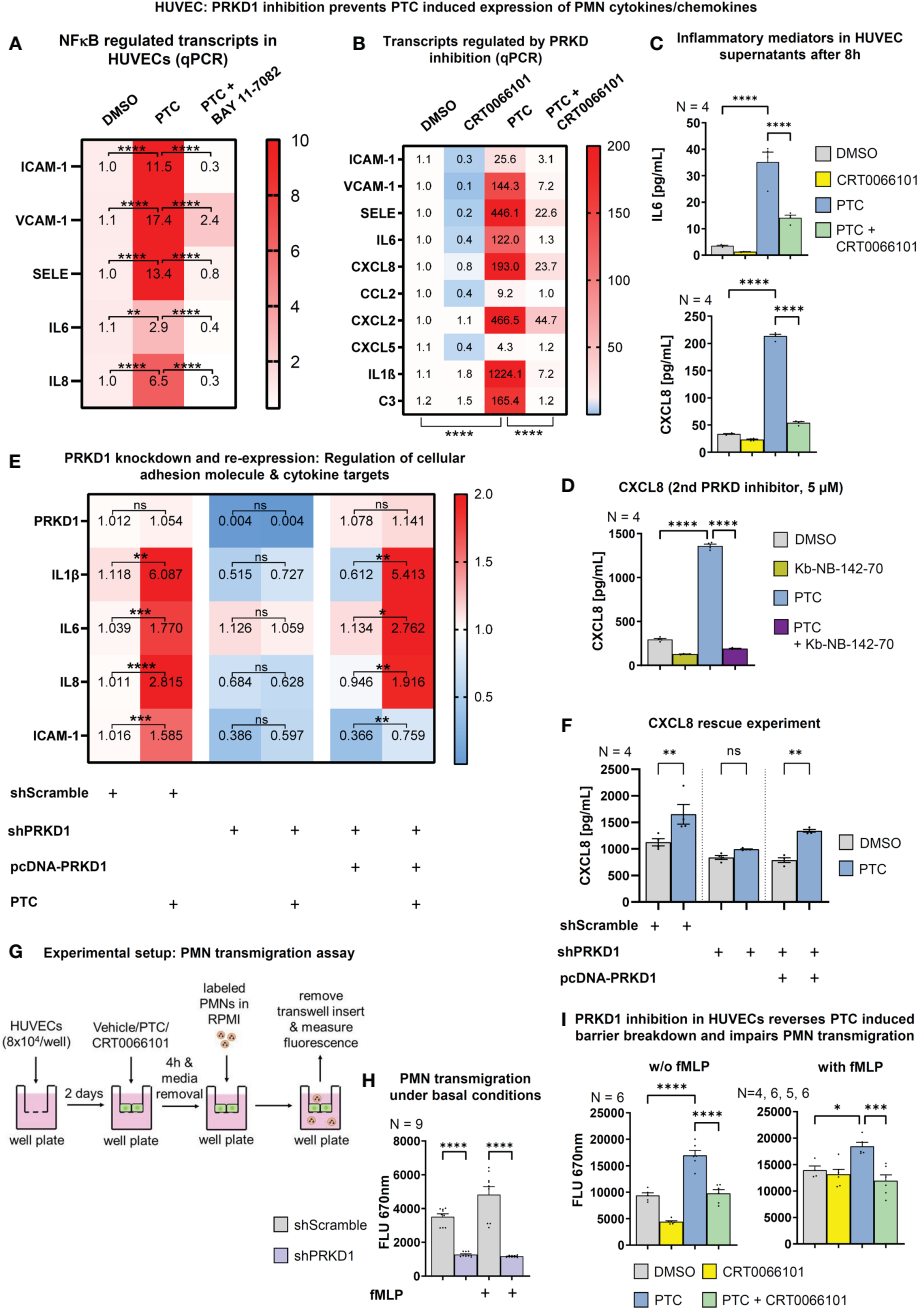
PRKD modulates expression of cellular adhesion molecules and inflammatory targets *via* NFκB. **(A)** Transcript levels of ICAM-1, VCAM-1, SELE, IL6 and CXCL8 in HUVECs after incubation with PTC and NFκB inhibitor BAY 11-7082 (10 µM) for 8 h as measured by qPCR. **(B)** qPCR to determine transcript levels of neutrophil relevant cellular adhesion molecules, cytokines and chemokines in HUVECs after treatment with PTC and CRT0066101 for 8 h. **(C)** Expression of IL6 and CXCL8 on protein level in HUVEC supernatants after 8 h incubation with PTC and CRT0066101 as measured by ELISA. **(D)** Exemplary expression levels of CXCL8 in HUVECs after incubation with PTC and Kb-NB-142-70 as measured by ELISA. **(E)** Transcript levels of IL1β, IL6, CXCL8 and ICAM-1 in HUVEC cells upon knockdown and re-expression of PRKD1 following PTC exposure measured by qPCR. Knockdown and re-expression of PRKD1 to endogenous levels was verified by qPCR (row 1). **(F)** CXCL8 levels in supernatants of **(E)** as detected by ELISA. **(G)** Flowchart of the transmigration assay procedure used for **(H)** and **(I)**. **(H)** Transmigration of fMLP stimulated PMNs through HUVEC monolayers stabilized by PRKD1 knockdown measured 90 min after PMN addition by fluorescence scanning of CellTracker-labeled PMNs using a Tecan M-200 plate reader. **(I)** Transmigration of fMLP stimulated PMNs across HUVEC monolayers that were treated with PTC and CRT0066101 for 8 h. Assays were performed as described in **(G)**. N-numbers indicate the number of independent samples. Statistical tests: **(A–I)** One-way ANOVA with multiple comparison post-test. *P < 0.05; **P < 0.01; ***P < 0.001, ****P < 0.0001; ns: no significant difference.

NFκB also controls expression of important post-traumatic pro-inflammatory mediators e.g., IL6 and CXCL8 (43, 44). Indeed, the NFκB inhibitor BAY 11-7082 (45) impaired the PTC-induced increase in IL6, CXCL8 and of all three cellular adhesion molecule transcripts **(** **Figure 6A** **)**. In addition, important pro-inflammatory, neutrophil-relevant cytokines/chemokines, such as IL6, CXCL8, CXCL2, CXCL5, C-C-chemokine ligand 2 (CCL2), IL1β, but also C3 were upregulated by PTC, and this upregulation was reverted by CRT0066101 **(** **Figure 6B** **)**. In most cases, the PRKD inhibitor even reduced basal transcript levels. This is of relevance, as CXCL2/5/8 (46–48) and CCL2 (49) act as neutrophil chemoattractants and IL1β as well as IL6 have pro-inflammatory functions (50, 51). We have also quantified release of IL6 and CXCL8 by HUVEC cells using ELISAs, which demonstrated the same regulation that could be further corroborated at the example of CXCL8 using the second PRKD inhibitor Kb-NB-142-70 **(** **Figures 6C, D** **)**. The concurrent regulation of the transcripts IL6, CXCL8, IL1β and ICAM-1 could also be demonstrated by knockdown and re-expression of PRKD1. Depletion of PRKD1 in HUVECs reverted the upregulation of the respective transcripts upon PTC stimulation and re-expression of PRKD1 in the knockdown cells reestablished a significant up-regulation upon PTC treatment **(** **Figures 6E**, **S1H** **)**. Of note, we were able to titrate PRKD1 expression from complete knockdown back to endogenous levels, providing clear evidence for regulation of the transcripts downstream of PRKD1. In addition, we also quantified CXCL8 secretion by ELISAs as part of the same experiments with congruent results **(** **Figures 6F**, **S1H** **)**.

Thus, our data demonstrate that activation of PRKD1 in endothelial cells downstream of PTC is implicated in the regulation of cellular adhesion molecules as well as neutrophil-relevant cytokines/chemokines, and it critically destabilizes the endothelial barrier. Therefore, we determined whether trans-endothelial migration of neutrophils would be affected by measuring the passage of fMLP stimulated PMNs across differentiated confluent HUVEC monolayers. PRKD1 depletion significantly impaired PMN migration with and w/o a chemotactic fMLP gradient after 90 min **(** **Figure 6G** **)**. In addition, we have pretreated HUVEC cells for 4 h with PTC and/or CRT0066101, before performing transendothelial migration assays against fMLP gradients. Here, CRT0066101 reverted increased PMN migration upon PTC treatment, supporting our previous molecular findings **(** **Figures 6H, I**, **S1H** **)**.

### 3.6 Concerted action of endothelial cytokines and sEVs promotes LTB_4_ biosynthesis in PMNs

**Figure 7:**
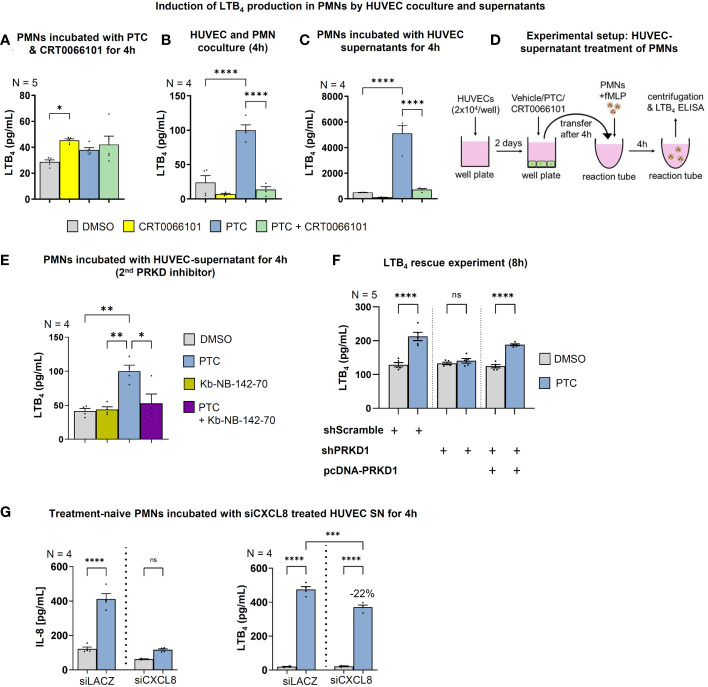
Endothelial PRKD regulates LTB_4_ production in PMNs. LTB_4_ concentrations in the supernatants of fMLP stimulated PMNs as measured by ELISA that were incubated: **(A)** with PTC and CRT0066101 for 4 h, **(B)** PTC and CRT0066101 treated HUVEC cells in coculture under equal conditions for 4 h, **(C)** treated with supernatants of HUVECs that were incubated with PTC and CRT0066101 for 4 h. **(E)** LTB_4_ concentrations in supernatants of fMLP stimulated PMNs that were treated with the supernatant of HUVECs incubated with PTC and/or Kb-NB-142-70 for 4 h. **(F)** LTB_4_ concentrations in supernatant of fMLP stimulated PMNs incubated with supernatant of HUVEC cells upon PRKD1 knockdown and re-expression (Figure 6E, S1H) as detected by ELISA. **(G)** Left-hand side: LTB_4_ concentrations in the supernatant of fMLP activated PMNs that were incubated with supernatant from siCXCL8 or siLACZ (control) treated HUVEC cells for 4 h, as measured by ELISA. Right-hand side: CXCL8 concentrations in HUVEC supernatant after treatment with siRNAs. N-numbers indicate the number of independent samples. Statistical tests: A-F) One-way ANOVA with Tukey’s multiple comparison post-test. *P < 0.05; **P < 0.01; ***P < 0.001, ****P < 0.0001; ns: no significant difference. **(D)** Experimental setup of the HUVEC supernatant treatment to initiate LTB4 production in PMNs used in 7C..

The extent of the post-traumatic immune response is critically dependent on the self-organized establishment of secondary chemotactic gradients by neutrophils. After extravasation and chemotaxis towards injuries, neutrophil behavior switches to highly coordinated unidirectional motion and clustering at wound cores (11, 52). This swarming process relies on paracrine release of the lipid attractant LTB_4_ (11). Yet, it currently remains unclear how neutrophil activation and chemoattractant synthesis are coordinated in the context of intercellular crosstalk with the endothelium. Since LTB_4_ biosynthesis (53) in neutrophils can be stimulated by chemokines, such as CXCL8 (54), we wondered whether endothelial cells and the PRKD1 signaling axis would also contribute to the regulation of secondary LTB_4_ gradients. Moreover, we have recently shown that post-traumatic endothelial sEVs are vital propagators of the inflammatory response after traumatic injury (14), and PRKD1 can quantitatively and qualitatively alter sEV secretion (13). We therefore performed *in vitro* coculture studies with PMNs and HUVEC cells to investigate intercellular communication. LTB_4_ production by PMNs alone was not dependent on PTC treatment or PRKD activity, respectively **(** **Figure 7A** **)**. However, upon coculture of PMNs with HUVECs, LTB_4_ generation was significantly increased by PTC stimulation. This increase was prevented by CRT0066101 coincubation, as measured by ELISAs in culture supernatants **(** **Figure 7B** **)**. Of note, these effects were even further potentiated by more than 50-fold when HUVEC cells were treated with PTC/CRT0066101 separately, and supernatants were transferred to fMLP activated PMNs to induce LTB_4_ synthesis **(** **Figures 7C, D** **)**. Thus, some components in the PTC treated supernatants of HUVEC cells robustly induced LTB_4_ production by fMLP activated PMNs, which was impaired upon PRKD inhibition. To validate this regulation for PMNs, we first excluded that the HUVEC cell culture supernatants contained LTB_4_ **(** **Figure S1I** **)**. In addition, we confirmed increased LTB_4_ production by supernatants upon PTC stimulation of HUVEC cells and inhibition by coincubation with the second PRKD inhibitor Kb-NB-142-70, as well as after knockdown of PRKD1, which was rescued by PRKD1 re-expression **(** **Figures 7E, F**, **S1H** **)**. Since the HUVEC cells release large amounts of additional CXCL8 upon PTC stimulation **(** **Figure 6C** **)**, we wondered whether this would contribute to the increase in LTB_4_ generation by PMNs following incubation with PTC stimulated HUVEC supernatants. To this end, we have initiated a depletion of CXCL8 from HUVEC cells prior to induction with PTC by siRNAs. As shown in **Figure 7G** (left side), we were able to strongly impair CXCL8 release into HUVEC supernatants upon PTC stimulation. However, LTB_4_ production was only partially inhibited by CXCL8 knockdown by 22% (**Figure 7G**, right side). Thus, additional components in the HUVEC supernatants are likely to be involved in triggering the major increase in LTB_4_ biosynthesis of PMNs.

**Figure 8:**
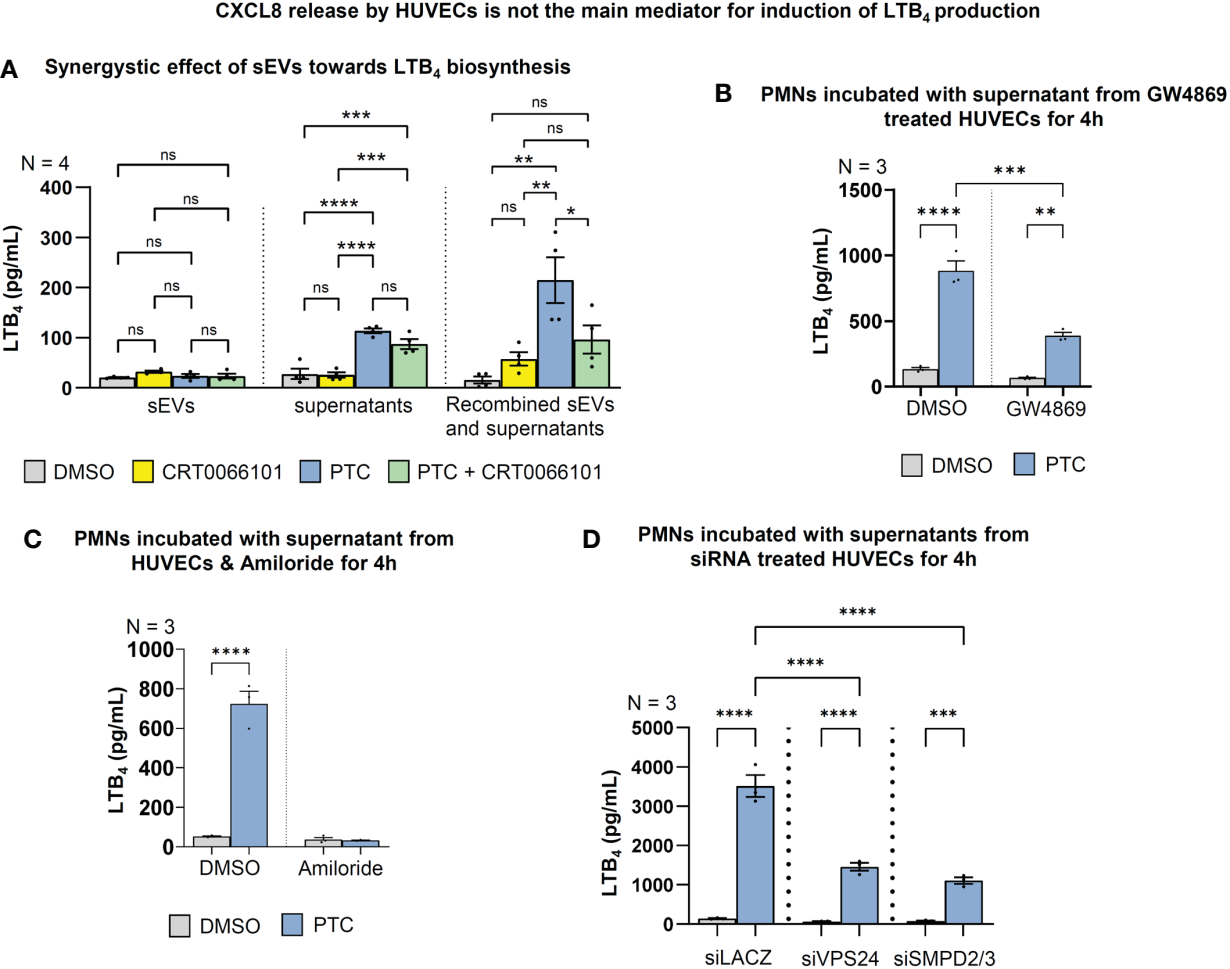
LTB_4_ production in PMNs is regulated by HUVEC-sEVs and soluble factors. **(A)** LTB_4_ concentrations in the supernatant of fMLP stimulated PMNs after incubation with 100.000g fractionated HUVEC supernatant +/- sEVs for 4 h, as detected by ELISA. **(B)** LTB_4_ concentrations in supernatant of fMLP stimulated PMNs that were incubated with supernatant of GW4869 treated HUVECs (10 µM) to block sEV biogenesis. **(C)** LTB_4_ concentrations in supernatant of fMLP stimulated PMNs that were incubated with the supernatant of HUVECs & Amiloride (2 mM) to block sEV uptake by macropinocytosis. **(D)** LTB_4_ concentrations in supernatant of fMLP stimulated PMNs that were treated with the supernatant of HUVECs upon siRNA mediated knockdown of VPS24 and SMPD2/3 for 48 h followed by PTC stimulation for 4 h. N-numbers indicate the number of independent samples. Statistical tests: A-D) One-way ANOVA with Tukey’s multiple comparison post-test. *P < 0.05; **P < 0.01; ***P < 0.001, ****P < 0.0001; ns: no significant difference.

To evaluate whether soluble factors or intercellular communication by EVs are responsible for triggering LTB_4_ synthesis in PMNs, we separated HUEVC supernatants into the respective fractions using 100,000g ultracentrifugation. Indeed, ultracentrifuged supernatants with soluble factors from PTC treated HUVECs significantly increased LTB_4_ release by PMNs, and the respective supernatants from PTC & CRT0066101 cotreated HUVECs partially reversed LTB_4_ synthesis. Interestingly, the EV pellet fraction alone barely enhanced LTB_4_ release, and a PRKD-dependent regulation of LTB_4_ was not detectable. However, when the respective supernatant and pellet EV fractions were recombined, LTB_4_ biosynthesis was prominently increased beyond the levels achieved by soluble factors, alone. The same increase in LTB_4_ synthesis was not observed in the PTC & CRT0066101 cotreatment group **(** **Figure 8A** **)**. Thus, both secreted soluble factors and EVs from endothelial cells are likely required to work in concert for efficient LTB_4_ biosynthesis by PMNs. To corroborate involvement of EVs, and in particular sEVs, described as vital regulators of post-traumatic inflammation (14), we treated HUVEC cells with PTC and the sEV biogenesis inhibitor GW4869, which blocks ceramide synthesis through inhibition of neutral sphingomyelinase (nSMase) (14, 55). The respective supernatants were harvested and incubated with PMNs for LTB_4_ production experiments **(** **Figure 8B** **)**. Vice versa, we have also employed an inhibitor of macropinocytosis (Amiloride) to block this major route for sEV uptake (56), in order to impair the transfer of HUVEC derived PTC-sEVs to PMNs **(** **Figure 8C** **)**. Again, both approaches significantly impaired LTB_4_ production upon treatment with PTC supernatants and therefore support modulation of LTB_4_ biosynthesis in PMNs *via* endothelial cell derived sEVs **(** **Figures 8B, C** **)**. These data were further corroborated by knockdown studies using siRNAs to deplete the essential endosomal-sorting-complex-required-for-transport (ESCRT)-III component VPS24 to impair ESCRT-dependent sEV biogenesis and the nSMAses, SMPD2/3 to inhibit ESCRT-independent sEV release (57) from HUVEC cells **(** **Figures 8D**, **S1F–G** **)**. In line with the inhibitor experiments, this strategy also strongly impaired LTB_4_ production in PMNs upon incubation with HUVEC derived PTC-supernatants. This suggests a vital role for post-traumatic HUVEC-sEVs in LTB_4_ synthesis by PMNs, which further requires secretory signals, such as endothelial CXCL8 for execution.

### 3.7 Inhibition of PRKD activity in endothelial cells enhances sEV secretion and impairs packaging of the key LTB_4_ biosynthesis enzyme LTA_4_ hydrolase in sEVs

**Figure 9:**
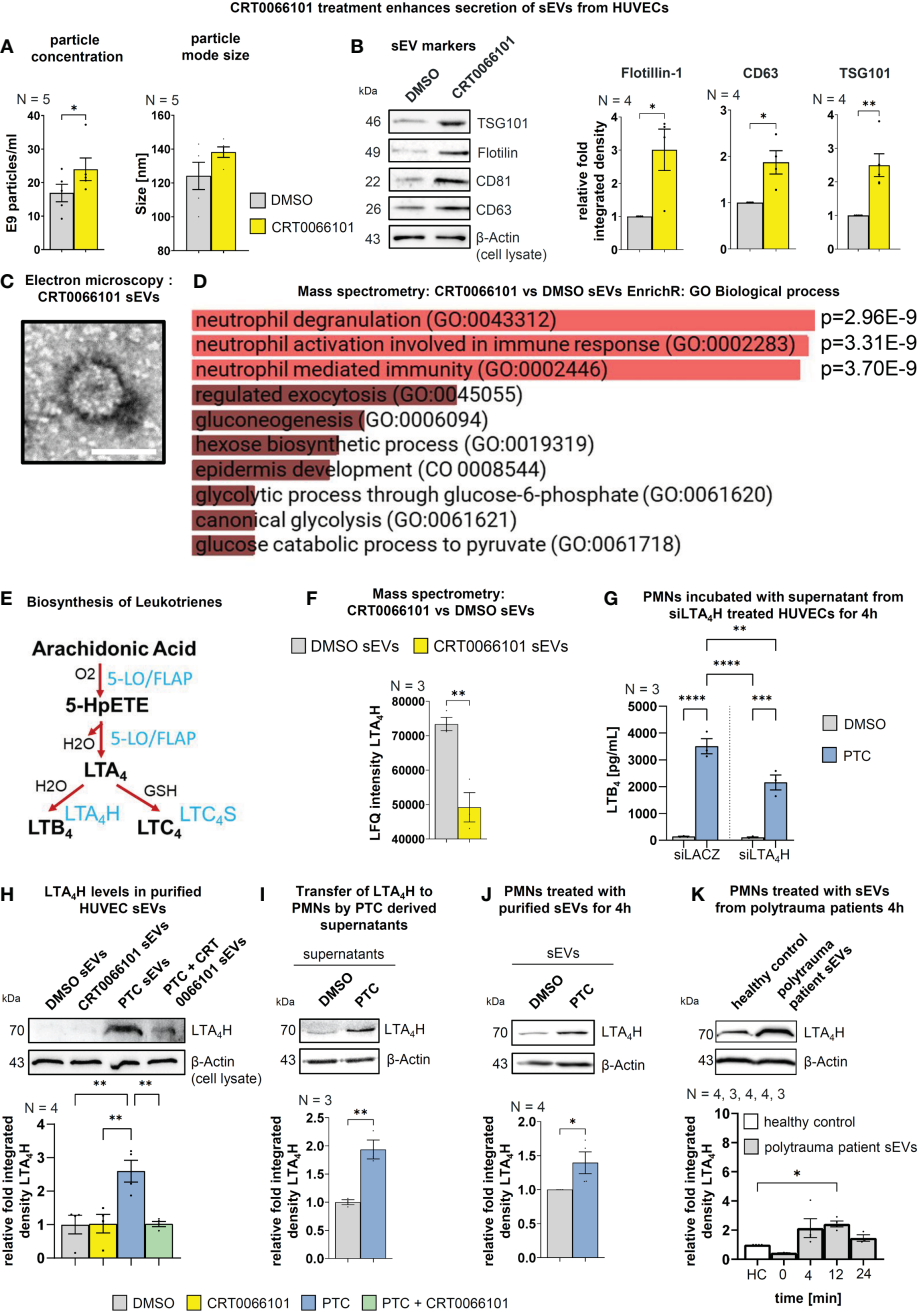
Endothelial PRKD quantitatively and qualitatively alters sEV release resulting in increased transfer of LTA_4_H to PMNs. **(A)** Nanoparticle tracking analysis (NTA) of purified sEVs from HUVEC cells treated with CRT0066101 or DMSO for 16 h. Left-hand side: Particle concentrations, Right-hand side: Average mode size of sEVs **(B)** Left-hand side: Western blot of sEV markers in purified sEVs from HUVECs treated as indicated for 16 h. Right-hand side: Quantification of sEV markers normalized to β-Actin levels in total cell lysates (TCLs) to compensate for cell numbers. **(C)** Exemplary transmission electron microscopy image of uranyl-shaded CRT0066101-sEVs. Scale 50 nm. **(D)** GO Biological process EnrichR meta-analysis of mass spectrometry data derived from CRT0066101- vs. DMSO-sEVs. **(E)** Scheme of leukotriene (LT) biosynthesis: The polyunsaturated fatty acid arachidonic acid (AA) is oxygenated by 5-lipoxygenase (5-LO) (activation by FLAP) to generate 5-hydroperoxy eicosapentaenoic acid (5-HpETE). 5-LO also catalyzes the dehydration of 5-HpETE to the intermediate LTA_4_. LTA_4_ is combined with H_2_O to generate 5,12-dihydroxy molecule (LTB_4_) or with glutathione to produce LTC_4_. The respective reactions are mediated by LTA_4_ hydrolase (LTA_4_H) and LTC_4_ synthase (LTC_4_S). LTB_4_ promotes adherence, chemotaxis, swarming, aggregation, and activation of PMNs. **(F)** Mass spectrometry LFQ intensities for LTA_4_H from HUVEC DMSO- and CRT0066101-sEVs **(G)** LTB_4_ concentrations in fLMP stimulated PMN supernatant that were incubated with the supernatant of siLTA_4_H-treated HUVEC cells +/- PTC stimulation for 4 h. **(H)** LTA_4_H levels in purified HUVEC-sEVs upon treatment with PTC and CRT0066101, as detected by Western blot normalized to β-Actin in TCL. **(I)** LTA_4_H expression in PMNs upon incubation with PTC/DMSO stimulated HUVEC supernatant for 4 h. **(J)** LTA_4_H expression in PMNs treated with equal amounts of HUVEC derived sEVs purified after incubation of cells with DMSO or PTC. **(K)** LTA_4_H expression in PMNs after 4 h treatment with equal amounts of patient derived sEVs purified from patient blood, which was drawn 0 (shock room admittance), 4, 12 or 24 h after trauma. N-numbers indicate the number of independent samples. Statistical tests: **(A, B, F, I, J)** Two-tailed unpaired student’s t-test. **(D)** Ordinary ANOVA with Benjamini-Krieger multiple comparison post-test. **(G, H)** One-way ANOVA with Tukey’s multiple comparison post-test. *P < 0.05; **P < 0.01; ***P < 0.001, ****P < 0.0001; ns: no significant difference.

Having demonstrated the requirement for post-traumatic sEVs and PRKD signaling in HUVEC cells for LTB_4_ biosynthesis in PMNs, we next investigated how CRT0066101 would alter PTC induced sEV secretion. We have previously shown that PTC enhances the release of pro-inflammatory sEVs from HUVEC cells that carry cytokine and cellular adhesion molecule transcripts to propagate inflammation to other endothelial cells (14). Crosstalk of these sEVs with PMNs, however was not studied in detail. To identify HUVEC sEV cargos implicated in LTB_4_ production that are also changed by PRKD inhibition, we conducted a MISEV analysis (58) in combination with proteome profiling. In line with our previous work in cancer cells (13), inhibition of PRKD significantly increased sEV release, as measured by nanoparticle tracking analysis (NTA) as well as sEV protein markers **(** **Figures 9A, B** **)**. Presence of nanovesicles in samples was corroborated by transmission electron microscopy (TEM) **(** **Figure 9C** **)**. Interestingly, EnrichR meta-analysis (59) for GO terms of mass spectrometry (MS) data on the respective sEVs obtained from CRT0066101 treated HUVEC cells, demonstrated a significant enrichment of signatures associated with neutrophil degranulation, activation, and immunity **(** **Figure 9D** **)**. Moreover, we identified a downregulation of the vital leukotriene biosynthesis enzyme LTA_4_ hydrolase (LTA_4_H) **(** **Figure 9F** **)** in CRT0066101 derived HUVEC-sEVs. LTA_4_H is essential to catalyze the last step of LTB_4_ synthesis (53). We therefore tested whether depletion of LTA_4_H from HUVEC cells prior to sEV production would impact on LTB_4_ levels released by PMNs. Indeed, we show a significant reduction in LTB_4_ biosynthesis from PMNs upon LTA_4_H knockdown in HUVECs **(** **Figure 9G** **)**. Next, we determined LTA_4_H levels in HUEVC-sEVs upon PTC and/or CRT0066101 treatment. LTA_4_H was strongly increased in sEVs after PTC treatment, and this was reversed by PRKD inhibition **(** **Figure 9H** **)**. We verified the transfer of LTA_4_H to PMNs using PTC-treated HUVEC supernatants as well as the respective purified sEVs **(** **Figures 9I, J** **)**. Furthermore, the relevance of these findings for patients was corroborated by treating PMNs from healthy donors with plasma-sEVs isolated from polytrauma patients (injury severity score ≥ 16) or healthy probands and then determining the respective LTA_4_H levels in cell lysates. The plasma samples were obtained from four patients at the time of shock room admittance (T0) as well as 4, 12 and 24 h after hospitalization. Relative concentration and size distribution of sEVs after purification by size exclusion chromatography was measured by nanoparticle tracking analysis, as part of our previous study in Seibold et al., 2021 (14). Concerning transfer of LTA_4_H, we indeed demonstrated significantly higher levels of LTA_4_H expression in PMNs after incubation with polytrauma patient-sEVs when compared to healthy proband-sEVs for the 12 h timepoint, whereas LTA_4_H after 4 h was already increased and after 24h level decreased again from the 12 h peak. **(** **Figure 9K** **).** In conclusion, our data suggest that PRKD alters endothelial sEV cargo composition to induce LTB_4_ biosynthesis in PMNs *via* transfer of LTA_4_H, which was also confirmed for polytrauma patient-sEVs.

### 3.8 Endothelial cells coordinate LTB_4_ production through intercellular communication with PMNs and thereby facilitate PMN activation as well as PMN oxidative burst

**Figure 10:**
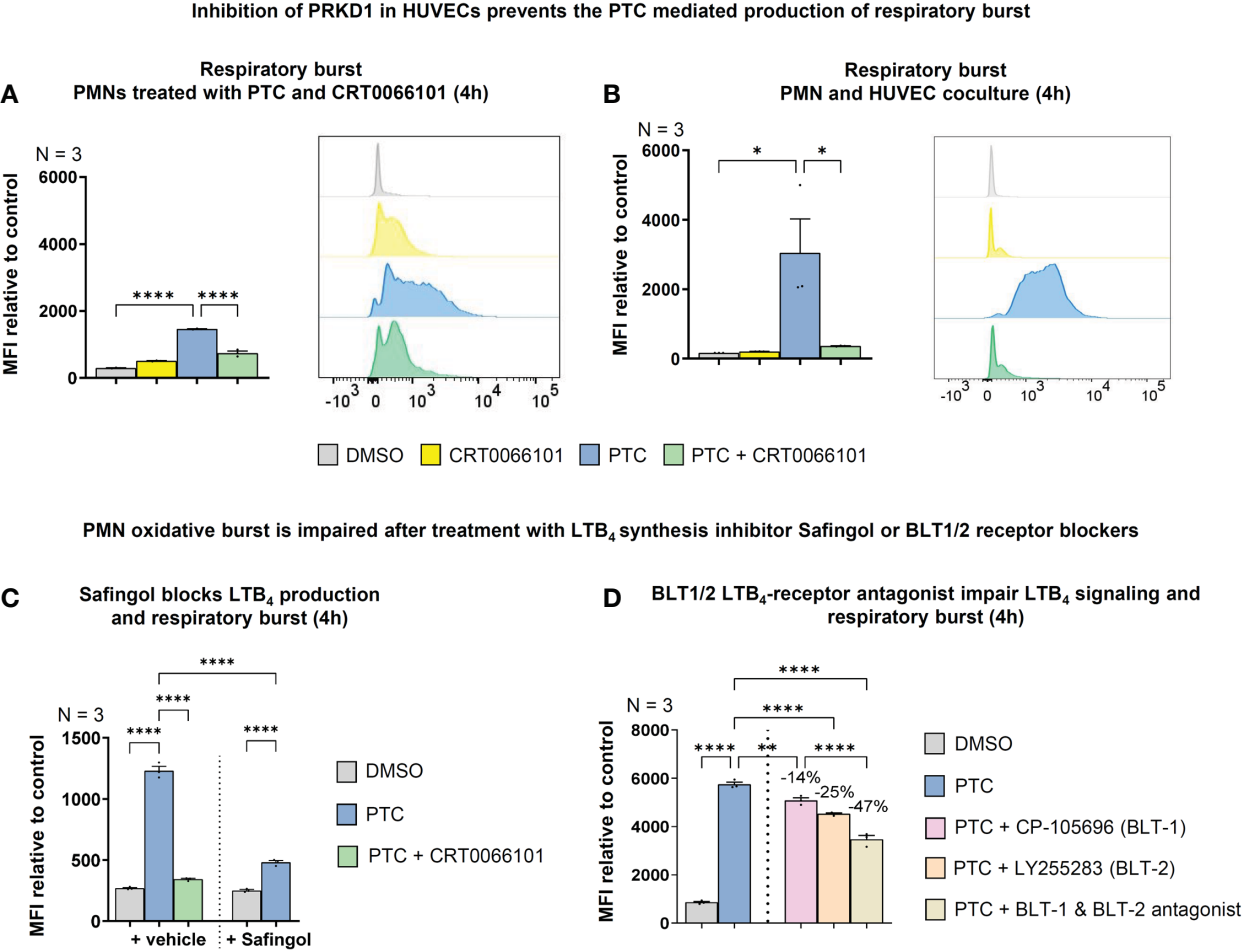
Endothelial PRKD amplifies PMN respiratory burst by stimulating LTB_4_ release from PMNs. **(A)** Left-hand side: Respiratory burst in fMLP activated PMNs treated with PTC and CRT0066101 for 4 h detected by dihydrorhodamine-123 upon conversion by reactive oxygen species using flow cytometry. Right-hand side: Exemplary flow cytometry histograms. **(B)** Left-hand side: Respiratory burst in fMLP stimulated PMNs upon coculture with HUVECs treated with PTC and CRT0066101 for 4 h. Right-hand side: Exemplary flow cytometry histograms. **(C)** Respiratory burst in fMLP stimulated PMNs incubated with HUVEC PTC derived supernatant and Safingol (10 µM, 4 h). **(D)** Respiratory burst in fMLP stimulated PMNs treated with HUVEC PTC derived supernatant combined with 1 μM CP-105696 (BLT1-receptor antagonist) & 1 μM LY255283 (BLT2-receptor antagonist). N-numbers indicate the number of independent samples. Statistical tests: A-D) One-way ANOVA with Tukey’s multiple comparison post-test. *P < 0.05; **P < 0.01; ****P < 0.0001; ns: no significant difference.

Besides a vital function as secondary chemoattractant, LTB_4_ released from PMNs also contributes to neutrophil activation (60). Therefore, we evaluated the neutrophil respiratory burst as a terminal readout for release of reactive oxygen species following activation (61). In line with a role of PTC in the coactivation of fMLP stimulated PMNs, the respiratory burst was slightly increased by PTC stimulation, but also by CRT0066101, when PMNs were measured on their own **(** **Figure 10A** **)**. However, in coculture with HUVECs, the respiratory burst was strongly increased by PTC treatment and this effect was substantially reversed upon PRKD inhibition with CRT0066101 **(** **Figure 10B** **)**. The respiratory burst of PMNs can be triggered by LTB_4_ (62). Therefore, we blocked LTB_4_ biosynthesis by the inhibitor Safingol (63) in PMNs, which prevented the increase in respiratory burst by PTC derived HUVEC supernatants **(** **Figure 10C** **)**. To corroborate these findings, we have used CP-10569 & LY255283 (1 µM) to inhibit the high and low affinity LTB_4_ receptors BLT1/2 (64), respectively, thus blocking upstream activation of PMNs. Also, these inhibitors significantly impaired the PTC supernatant mediated respiratory burst alone, but more efficiently in combination, suggesting that both pathways are utilized **(** **Figure 10D** **)**.

In summary these data indicate that activation of the PRKD1 axis downstream of post-traumatic inflammatory mediators reprograms intercellular communication of endothelial cells to control the magnitude of the post-traumatic immune response from PMNs *via* LTB_4_ and the induction of the PMN respiratory burst.

## 4 Discussion

**Figure 11:**
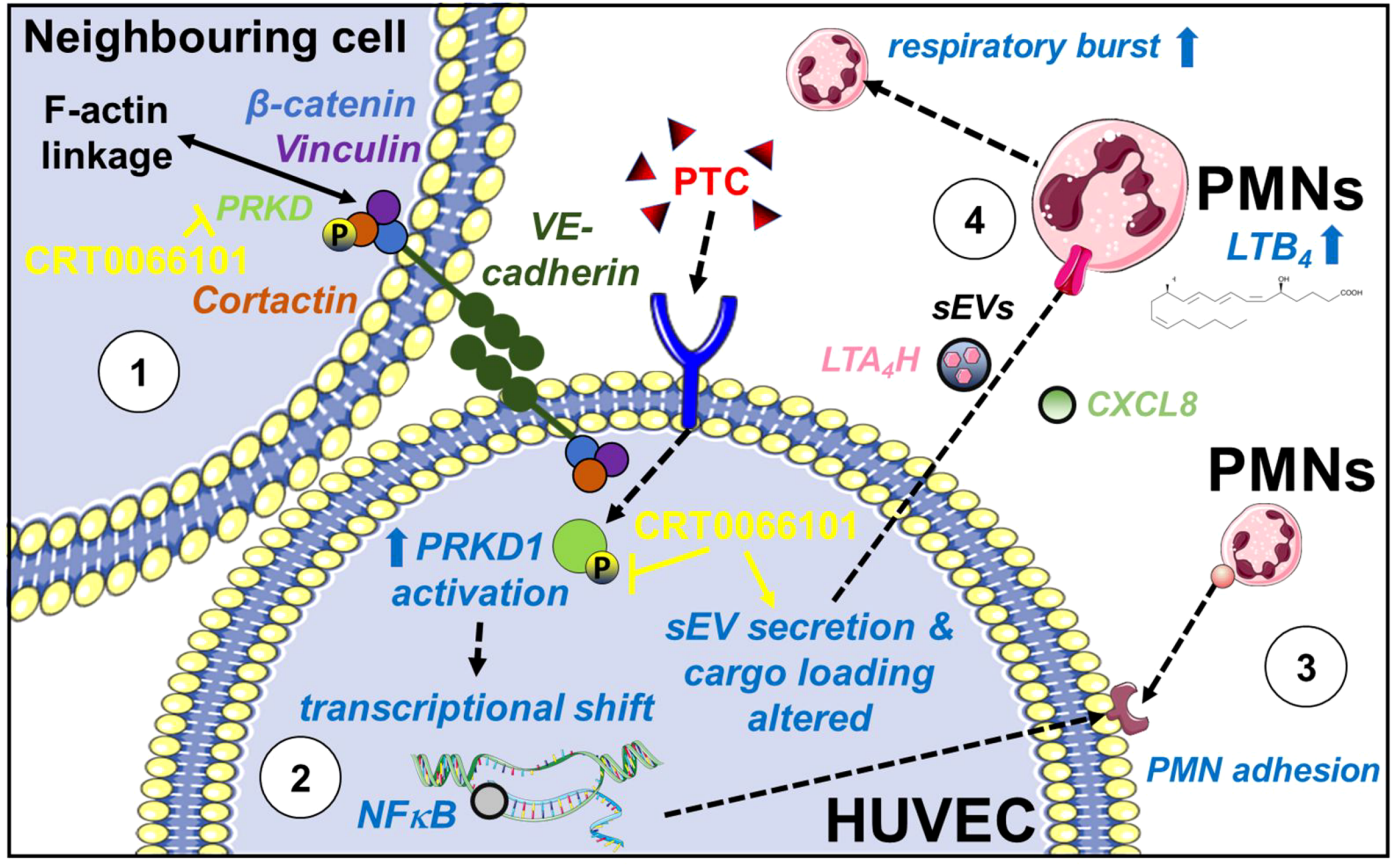
Role of PRKD in post-traumatic hyperinflammation. PRKD1 is activated by a pro-inflammatory stimulus e.g., polytrauma cocktail (PTC: IL1β, IL6, CXCL8, C3a and C5a-des-Arg) in endothelial cells resulting in (1) destabilization of the endothelial barrier caused by abrogating F-actin linkage of VE-cadherin adhesion complexes *via* its kinase substrate Cortactin and upregulation of ROCK1 expression. Inhibition of PRKD activity reverts this phenotype. (2) transcriptional upregulation of pro-inflammatory cytokines and chemokines (IL6, IL1β, CXCL8, CXCL5) and cellular adhesion molecules (ICAM-1, VCAM-1 and SELE) *via* the NFκB pathway. (3) enhanced PMN adhesion/interaction due to increased surface expression of ICAM-1, VCAM-1 and E-Selectin, driving transmigration of PMNs across the endothelium. (4) reprogramming of endothelial sEV cargos (transfer of LTA_4_H) and release of pro-inflammatory secreted factors (e.g., CXCL8) to prime and promote LTB_4_ biosynthesis in PMNs. LTB_4_ is known to cause swarming behavior of PMNs thus determining the extend of the host’s inflammatory response. Moreover, LTB_4_ mediates activation of the PMN respiratory burst.

Post-traumatic hyperinflammation is caused by an uncontrolled, exaggerated innate immune response (6). Following a traumatic injury, first DAMPs are generated and then cytokines are released, which activate neutrophils (PMNs) and endothelial cells. Originally PMNs are thought to follow chemotactic gradients, like fMLP (9) towards the site of an injury and then switch their behavior to self-organized swarming by attracting, clustering and activating additional PMNs through secondary lipid gradients, like LTB_4_ (11). In addition, it has been documented for several years that different cell types cooperate to produce Leukotrienes e.g., by transfer of arachidonic acid intermediates (65). This phenomenon is known as transcellular Leukotriene biosynthesis (65). Our current study indicates that these processes are indeed much more complex and require a coordinated intercellular communication between endothelial cells and PMNs by direct cell-cell contacts, secreted cytokines and post-traumatic sEVs from endothelial cells, which critically define the magnitude of neutrophil activation **(** **Figure 11** **)**. Earlier studies have postulated an inhibitory effect of the endothelium on LTB_4_ production by PMNs, some have even suggested a vital role in the reversal of transendothelial migration (66). However, to induce strong LTB_4_ production by PMNs, our data indicate that activated endothelial cells release pro-inflammatory sEVs containing the LTB_4_ biosynthesis enzyme LTA_4_H **(** **Figures 9H–J** **)**. Uptake of theses sEVs primes PMNs for LTB_4_ production. Yet, biosynthesis is only induced when additional soluble factors are present **(** **Figure 8A** **)**. Since activated endothelial cells also release large amounts of CXCL8, which is involved in the induction of LTB_4_ biosynthesis by PMNs (54), we suggest that additional secretory communication from endothelial cells further determines the extent of PMN activation **(** **Figure 7G** **)**. The relevance of intercellular communication by sEVs was further corroborated using serum-sEVs isolated from polytrauma patients, which likewise increased LTA_4_H expression levels in PMNs **(** **Figure 9K** **)**. Interestingly in polytrauma patients, also high CXCL8 concentrations have been associated with severe adverse events. CXCL8 was correlated with development of ARDS and multi organ failure (MOF) as well as with fatal outcome (67–69). In line, CXCL8 was suggested as a marker to identify patients at risk to develop ARDS and MOF (69). Although we did not aim to characterize new biomarkers in our current study, our data suggest that serum-sEV-LTA_4_H levels and CXCL8 concentrations should be investigated in more detail as a putative marker combination to determine the magnitude of post-traumatic inflammation and risk for severe complications.

Having investigated the underlying molecular mechanisms that govern LTB_4_ biosynthesis, we have demonstrated the involvement of endothelial PRKD1 in mediating qualitative and quantitative changes in sEV cargo that result in the release of LTA_4_H-positive sEVs **(** **Figures 9A–F** **)**. We have shown that PRKD1 activation in endothelial cells is induced *in vitro* by a cytokine and anaphylatoxin cocktail (PTC) as well as after severe trauma in mice **(** **Figures 1A–D** **)**. In addition, activation of the PRKD1-NFκB signaling axis downstream of PTC promoted enhanced expression and release of pro-inflammatory mediators, like CXCL8 and IL6 as well as the upregulation of the endothelial adhesion molecules, ICAM-1, VCAM-1 and E-selectin **(** **Figures 5D**, **6C** **)**. This is in line with the function of PRKD1 as an upstream regulator of NFκB following stress, and in particular oxidative stress exposure (27). Moreover, another study has reported that PRKD1 mediated NFκB signaling was responsible for controlling inflammatory processes *in vivo* e.g. immune cell infiltration, inflammation and IL6 release, which were abrogated by employing a PRKD inhibitor in a model of experimental pancreatitis in rats (70). Besides, activation of PRKD1 also critically destabilized the endothelial barrier in our experiments by dissociating the actin linkage and impairing stability of VE-cadherin adherens junctions **(** **Figures 2C–F**, **4A, B** **)**. These data are is in line with our previous work in Sroka et al., 2016 (21), where we have unravelled molecular mechanisms based on phosphorylation of the PRKD kinase substrate Cortactin, controlling F-actin linkage for E-cadherin complexes. Interestingly, endothelial barrier stabilization upon PRKD inhibition was very efficient and comparable to inhibiting the major regulator of barrier integrity ROCK1 using the Y27632 inhibitor (35). Both pathways are controlled by RhoGTPase activation, which destabilizes epithelial or endothelial barrier integrity (21, 71). Moreover, barrier destabilization downstream of PRKD1 was sufficient to potentiate neutrophil transendothelial passage *in vitro* **(** **Figures 6G–I** **)**. We have further shown important functions of PRKD in neutrophils (9). PRKD1 activation in response to fMLP balanced the extent of actin remodelling and cellular deformability by phosphorylation of the cofilin-phosphatase Slingshot-2L (9). Thus, activation of PRKD1 in endothelial cell and PMNs in the context of trauma is a vital signaling node that controls and shapes the extent of post-traumatic inflammation at multiple levels. Therefore, PRKD inhibitors may be a promising option to limit post-traumatic hyperinflammation.

In this regard however, our study also has some limitations. Even though we have unravelled a major function of PRKD1 in endothelial cells for PMN-driven post traumatic inflammation *in vitro*, and we have described molecular mechanisms (**Figure 11** **)**, further evidence in mice is currently lacking. Thus, in depth mouse studies, concerning intervention with PRKD inhibitors would be desirable, especially since CRT0066101 has already been applied to limit growth of different tumor entities in mice and was reasonably well tolerated (19, 72, 73). In these experiments, CRT0066101 has shown the potential to impact on tumor cell differentiation, proliferation as well as cell motility (19), e.g., by controlling actin remodelling (74, 75). While desirable in the context of tumor therapy, these effects may cause adverse events during therapeutic intervention in post-traumatic inflammation. We have previously shown that PRKD inhibition by CRT0066101 can critically impair neutrophil cell migration and transendothelial passage (9). Thus, the time point of CRT0066101 deployment as well as the dosage must be chosen cautiously, as not to completely abrogate the post-traumatic immune response. Ideally, CRT0066101 would be administered in a way that blunts both post-traumatic sEV release with pro-inflammatory cargo, as well as cytokine secretion, and leukotriene biosynthesis *via* the pathway discovered in this study. To this end, we have shown that the peak of post-traumatic sEV secretion in mice occurred at about 4 h after injury (14). Therefore, intervention must be timed early, as shown for the sEV biogenesis inhibitor GW4869, with an injection 15 min post trauma to prevent the release of sEVs with pro-inflammatory cargo molecules (14). Early intervention at this time point however can also abrogate initial infiltration of injury sites by neutrophils and other immune cells, which is required to mount a proper post-traumatic immune response (6). In contrast, the peak of LTB_4_ production usually occurs later after trauma. In a polytrauma porcine model a notable increase in plasma LTB_4_ concentration was detected 72 h post-injury (76). To counteract hyperinflammation, the whole spectrum of PRKD mediated effects must be considered, e.g., post-traumatic sEV secretion, barrier breakdown, cytokine as well as LTB_4_ release. Therefore, intervention experiments with CRT0066101 in mice must be designed carefully to determine a suitable therapeutic window and duration for inhibitor application. These experiments are planned as part of a future in-depth study to determine treatment parameters. This is of particular interest since we have already confirmed the transfer of LTA_4_H to PMNs for polytrauma patient-sEVs **(** **Figure 9K** **)**.

In conclusion, we demonstrate an important role for intercellular communication by endothelial derived sEVs in propagating inflammatory signals after traumatic injury to the larger endothelium (14), but also to PMNs, summarized in **Figure 11**. Our data provide mechanistic insights into the intercellular crosstalk of the endothelium with PMNs at multiple levels and suggest that our findings concerning the transfer of sEV derived endothelial LTA_4_H in combination with CXCL8 should be investigated as putative biomarkers. This may help predict and potentially treat a hyperinflammatory response or the onset of side effects, such as ARDS or MOF (67–69) after severe traumatic tissue injury in patients.

## Data availability statement

The raw data supporting the conclusions of this article will be made available by the authors, without undue reservation.

## Author contributions

TE and ThS conceived the project. TE, ThS designed and supervised the experiments. TE, ThS and JS interpreted results, and wrote the paper with input from all authors. JS, TS performed TxT animal experiments. MH-L and provided PT + HS & PT mouse lung section. MH-L and MK provided plasma of polytrauma patients. JS, TS, MM, MA, RS, JC, NS, JG, LS, TE performed *in vitro* experiments. All authors contributed to the article and approved the submitted version.
