# Supplementary material for: Endothelial Protein kinase D1 is a major regulator of post-traumatic hyperinflammation
Source: Front Immunol. 2023 Mar 2;14:1093022. doi: 10.3389/fimmu.2023.1093022 (PMC10017463; doi:10.3389/fimmu.2023.1093022)
Supplement: Supplementary file 1 [file DataSheet_1.pdf]

Supplementary Material

1 Supplementary Data

2 Supplementary Figures and Tables

2.1 Supplementary Figures

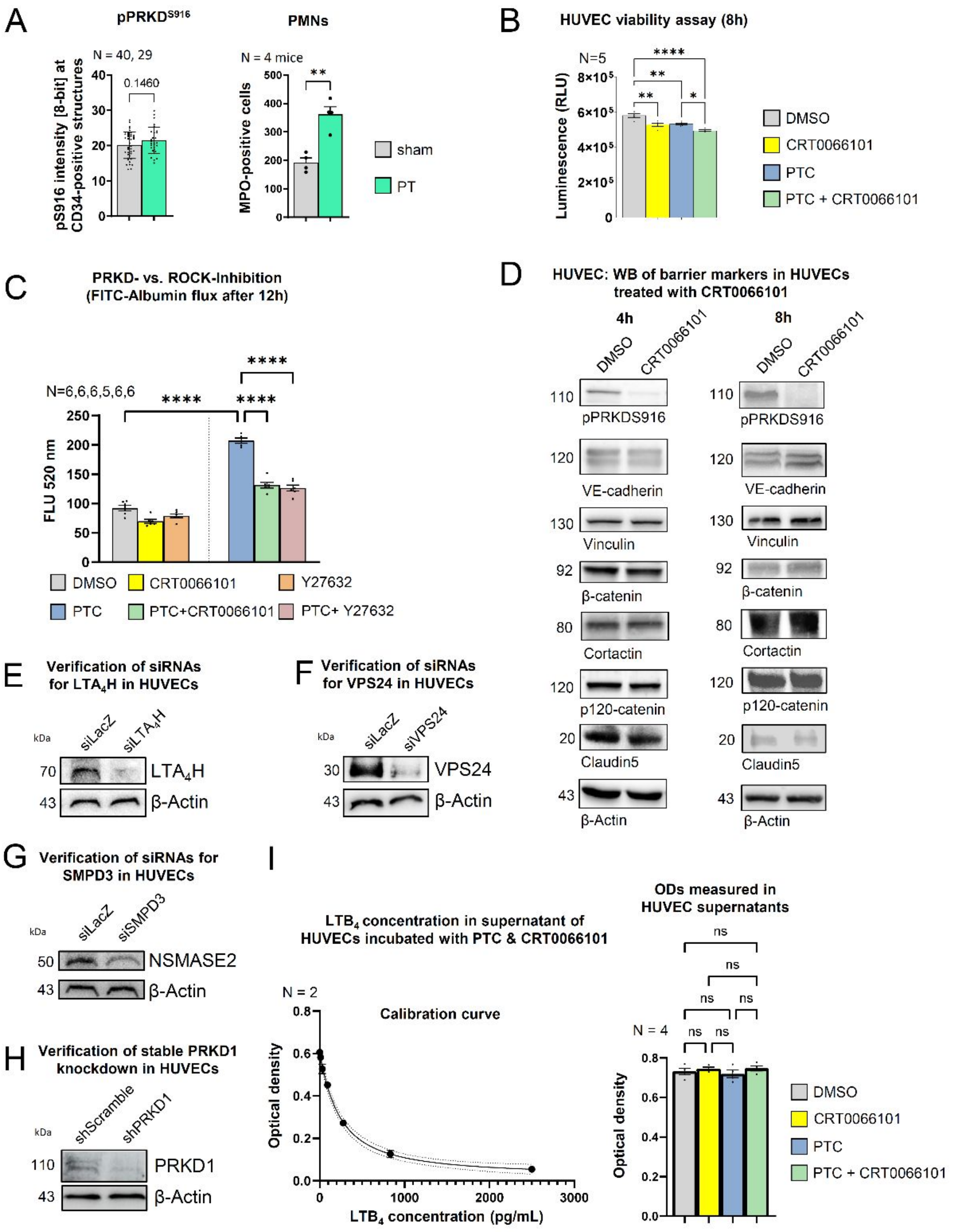

Supplemental Figure S1: (A) PRKD activation in lung microvascular endothelial cells and infiltration of PMNs into lungs of polytrauma mice (PT) (N=4). Images acquisition and analysis was performed as previously described for the corresponding samples of polytrauma mice with hemorrhagic shock in Figure 1D-E. **(B)** CellTiter-Glo 2.0 viability assay performed in HUVEC cells after incubation with PTC & CRT0066101 (5 µM) for 8 h. **(C)** Endothelial barrier stability of HUVECs treated with PTC, CRT0066101 (5 µM) and the ROCK-inhibitor Y27632 (10 µM) measured by FITC-albumin flux assay after 12 h. **(C)** Endothelial barrier stability of HUVECs treated with thrombin and CRT0066101 (5 µM) measured by FITC-albumin flux assay. **(D)** Western blot (WB) of barrier markers in HUVECs treated with CRT0066101 (5 µM) for 4 and 8 h. **(E)** Measurement of LTB_4 concentrations upon knockdown of LTA_4H in HUVECs as shown in Fig. 9G. The WB demonstrates the depletion of LTA_4H in an exemplary experiment. One of at least three experiments is shown. **(F)** Measurement of LTB_4 concentrations upon knockdown of VPS24 in HUVECs as shown in Fig. 8D. The WB demonstrates the depletion of VPS24 in an exemplary experiment. One of at least three experiments is shown. One of at least three experiments is shown. **(G)** Measurement of LTB_4 concentrations upon knockdown of SMPD3 (NSMASE2) in HUVECs as shown in Fig. 8D. The WB demonstrates the depletion of SMPD3 isoform in an exemplary experiment. One of at least three experiments is shown. **(H)** PRKD1-knockdown cells used in Figures 2B, 5F, 6E, 6F, 6H and 7E. Polyclonal stable knockdown cells for PRKD1 in HUVEC cells were generated by lentiviral transduction and puromycin selection. The WB demonstrates stable knockdown of PRKD1 in these cells. **(H)** HUVEC cells do not produce LTB_4 upon PTC treatment. The graphs depict the calibration curve for the ELISA measurement of LTB_4 and the observed OD_{450} for the measured HUVEC supernatants at the indicated conditions. N-numbers indicate the number of quantified junctions (1 junction per image, 3 independent stainings). Statistical tests: A) Two-tailed unpaired student’s t-test; B, C, I) One-way ANOVA with Tukey’s multiple comparison post-test *P<0.05; **P<0.01; ****P <0.0001; ns: no significant difference.
