# Supplementary material for: Endothelial Protein kinase D1 is a major regulator of post-traumatic hyperinflammation
Source: Front Immunol. 2023 Mar 2;14:1093022. doi: 10.3389/fimmu.2023.1093022 (PMC10017463; doi:10.3389/fimmu.2023.1093022)
Supplement: Supplementary file 2 [file Table_1.docx]

Supplementary Material

# Supplementary Data

## Supplementary Figures and Tables

## Supplementary Tables

Supplementary Table S1: Key resources used in this study

| **KEY RESOURCES** |  |  |
| --- | --- | --- |
| **REAGENT or RESOURCE** | **SOURCE** | **IDENTIFIER** |
| **Antibodies / staining agents** | | |
| anti-TSG-101 | GeneTex Inc. | GTX70255 |
| anti-Flotillin | Cell Signalling | 3253S |
| anti-CD81 | SantaCruz | sc-7637 |
| anti-CD63 | Santa Cruz | sc-5275 |
| anti-Myeloperoxidase | Abcam | ab9535 |
| anti-p120-catenin | SantaCruz | sc-13957 |
| anti-β-actin AC-15 | Sigma Aldrich | A1978 |
| anti-β-Catenin | BD Transduction Laboratories | 610154 |
| anti-VE-Cadherin | SantaCruz | sc-28644 |
| anti-Claudin-5 | SantaCruz | sc-374221 |
| anti-PRKD1 | Cell Signalling | D4J1N |
| anti-pPRKDS910/916 | Cell Signalling | 2051S |
| anti-pp65S536 | Cell Signalling | 3033S |
| anti-ROCK1 | Cell Signalling | 4035S |
| anti-VE-Cadherin | SantaCruz | sc-9989 |
| anti-Vinculin | Sigma Aldrich | V9264 |
| anti-Cortactin | SantaCruz | sc-11408 |
| anti-LTA4H | SantaCruz | sc-514465 |
| anti-NSMASE2 | SantaCruz | sc-166637 |
| anti-CHMP3 | SantaCruz | sc-166361 |
| anti-p65 | Cell Signalling | 8242 |
| anti-CD34 | Abcam | 8158 |
| anti-mouse IgG HRP | GE Healthcare Life Sciences | NA931V |
| anti-rabbit IgG HRP | GE Healthcare Life Sciences | NA934V |
| Alexa-Fluor 488/568/647 goat-anti-mouse | ThermoFisher Scientific | A11031 |
|  |  | A11001 |
|  |  | A21235 |
| Alexa-Fluor 488/568/647 goat-anti-rabbit | ThermoFisher Scientific | A11034 |
|  |  | A11011 |
|  |  | A21244 |
| Alexa-Fluor 647 Phalloidin | ThermoFisher Scientific | A22287 |
| **Antibodies for flow cytometry** | | |
| anti-(ICAM-1)-APC +Isotype | Miltenyi Biotec | 130-103-840 |
| anti-(VCAM-1)-FITC Isotype | Miltenyi Biotec | 130-104-124 |
| anti-(CD62E)-APC Isotype | Miltenyi Biotec | 130-105-468 |
| **Chemicals, Inhibitors, Peptides, and Recombinant Proteins** | |  |
| Thrombin | Sigma Aldrich | T6884 |
| GW4869 | Sigma Aldrich | D1692-5MG |
| CRT0066101 | Sigma Aldrich | SML1507 |
| kb-NB-142-70 | Sigma Aldrich | SML0525 |
| BAY 11-7082 | Sigma Aldrich | 196870 |
| Amiloride | Sigma Aldrich | A7410 |
| y-27632 | Sigma Aldrich | Y0503 |
| Safingol | Sigma Aldrich | 860488P |
| CP-105696 | Sigma Aldrich | PZ0363 |
| LY255283 | Cayman | 70715 |
| IL1ß | PeproTech | 200-01B |
| IL6 | PeproTech | 200-06 |
| CXCL8 | PeproTech | 200-08 |
| C3a | abbexa | abx066057 |
| C5a des-arg | Merck | 204902-30UG |
| **Critical Commercial Assays** | |  |
| human IL-6 ELISA kit | ThermoFisher Scientific | BMS213-2 |
| human IL-8/CXCL8 ELISA | R&D Systems | D8000C |
| LTB_4_ ELISA | R&D Systems | KGE006B |
| LTB_4_ ELISA | Enzo | ADI-901-068 |
| Respiratory Burst kit | Cayman | 601130 |
| **Experimental Models: Cell Lines** |  |  |
| HUVEC | ATCC | CRL-1730 |
| **Experimental Models: Organisms/Strains** |  |  |
| C57BL/6 | purchased from animal facility Ulm |  |
| **qPCR Primer human** |  |  |
| C3 | Qiagen | QT00089698 |
| CCL2 | Qiagen | QT00212730 |
| CXCL2 | Qiagen | QT00013104 |
| CXCL5 | Qiagen | QT00203686 |
| CXCL8 (IL-8) | Qiagen | QT00000322 |
| GAPDH | Qiagen | QT00079247 |
| ICAM-1 | Qiagen | QT00074900 |
| IL1b | Qiagen | QT00021385 |
| IL6 | Qiagen | QT00083720 |
| PRKD1 | Qiagen | QT00084847 |
| RHOB | Qiagen | QT00227409 |
| ROCK1 | Qiagen | QT00034972 |
| ROCK1 | Qiagen | QT00034972 |
| SELE (E-Selectin) | Qiagen | QT00015358 |
| TNF-alpha | Qiagen | QT00029162 |
| VCAM-1 | Qiagen | QT00018347 |
| **siRNAs** |  |  |
| siLTA4H | invitrogen | s8306 |
| siCXCL8 | invitrogen | s7327 |
| siIL6 | invitrogen | s7313 |
| SMPD3 | invitrogen | s30927 |
| VPS24 | invitrogen | s28474 |
| **shRNAs** |  |  |
| shScramble | Sigma-Aldrich | shc002 |
| shPRKD1 | Sigma-Aldrich | NM_002742.x-2978s1c1 |
| **Lentiviral packaging constructs** |  |  |
| psPAX2 | Addgene | #12260 |
| pMD2.G | Addgene | #12259 |
| **Mammalian expression constructs** |  |  |
| pcDNA3-PRKD1 | Hausser et al. 2002 |  |
